# Biophysically inspired model for functionalized nanocarrier adhesion to cell surface: roles of protein expression and mechanical factors

**DOI:** 10.1098/rsos.160260

**Published:** 2016-06-29

**Authors:** N. Ramakrishnan, Richard W. Tourdot, David M. Eckmann, Portonovo S. Ayyaswamy, Vladimir R. Muzykantov, Ravi Radhakrishnan

**Affiliations:** 1 Department of Bioengineering, University of Pennsylvania, Philadelphia, PA 19104, USA; 2 Department of Chemical and Biomolecular Engineering, University of Pennsylvania, Philadelphia, PA 19104, USA; 3 Department of Anesthesiology and Critical Care, University of Pennsylvania, Philadelphia, PA 19104, USA; 4 Department of Mechanical Engineering and Applied Mechanics, University of Pennsylvania, Philadelphia, PA 19104, USA; 5 Center for Targeted Therapeutics and Translational Nanomedicine, Institute for Translational Medicine and Therapeutics and Department of Pharmacology, School of Medicine, University of Pennsylvania, Philadelphia, PA 19104, USA; 6 Translational Research Center, The Perelman School of Medicine, University of Pennsylvania, Philadelphia, PA 19104, USA; 7 Department of Biochemistry and Biophysics, University of Pennsylvania, Philadelphia, PA 19104, USA

**Keywords:** functionalized nanocarrier, membrane biophysics, excess area

## Abstract

In order to achieve selective targeting of affinity–ligand coated nanoparticles to the target tissue, it is essential to understand the key mechanisms that govern their capture by the target cell. Next-generation pharmacokinetic (PK) models that systematically account for proteomic and mechanical factors can accelerate the design, validation and translation of targeted nanocarriers (NCs) in the clinic. Towards this objective, we have developed a computational model to delineate the roles played by target protein expression and mechanical factors of the target cell membrane in determining the avidity of functionalized NCs to live cells. Model results show quantitative agreement with *in vivo* experiments when specific and non-specific contributions to NC binding are taken into account. The specific contributions are accounted for through extensive simulations of multivalent receptor–ligand interactions, membrane mechanics and entropic factors such as membrane undulations and receptor translation. The computed NC avidity is strongly dependent on ligand density, receptor expression, bending mechanics of the target cell membrane, as well as entropic factors associated with the membrane and the receptor motion. Our computational model can predict the *in vivo* targeting levels of the intracellular adhesion molecule-1 (ICAM1)-coated NCs targeted to the lung, heart, kidney, liver and spleen of mouse, when the contributions due to endothelial capture are accounted for. The effect of other cells (such as monocytes, etc.) do not improve the model predictions at steady state. We demonstrate the predictive utility of our model by predicting partitioning coefficients of functionalized NCs in mice and human tissues and report the statistical accuracy of our model predictions under different scenarios.

## 1. Introduction

Design and optimization of affinity–ligand functionalized nanocarriers (NCs) for diagnostic and therapeutic purposes remains an active area of biomedical research [1]. Immense advances have been made on the design front resulting in a huge repertoire of NC configurations that encompass carriers as diverse as liposomes, inorganic particles, DNA cages, nanodiamonds and polymer-based formulations such as polymerosomes and nanogels [2–4]. Each of these formulations shows higher efficacy only in a limited context and specific tissues, and in the majority of the cases the reason for the conducive behaviour is not always evident. This is due to a lack of understanding of the fundamental principles governing the pharmacokinetics (PK) of the NC [3,4]. The understanding of how the delivery vehicle interacts with the target tissue and how the design parameters may be varied to reach optimal function still remains preliminary, because of the large dimensions of the design space and the interconnected nature of the parameter space, as well as the complex physiological and biochemical factors that govern NC binding [3–5]. Traditional PK models, which are often guided by heuristic principles or empirically determined functions are inadequate in the context of targeted drug delivery, because their parameters can seldom be tuned rationally, and can only be obtained through exhaustive *in vivo* experiments. In contrast to the empirical approaches, biophysical approaches can enhance the predictive value of such models, especially for newer classes of vectors for which the design/parameter space is large/high-dimensional.

The need for rational design in biomedicine has long been recognized, and efforts to devise a framework to describe the complex behaviour of NCs have been taken both from the experimental and theoretical viewpoints. A combinatorial approach involves the synthesis of multiple NC formulations, each with well-controlled chemical and physical properties, and uses high-throughput screening techniques to map the relationship between the formulation-type and efficacy [6]. Theoretical studies take a systems-based approach which includes cooperative binding, the physical state of the cell membrane and entropy as synergistic/competing factors that determine NC avidity. Frenkel and co-workers [7,8] have used statistical mechanics-based models and Monte Carlo (MC) simulations to determine receptor–ligand interaction parameters for selective targeting of NCs. Weikl and co-workers [9–11] have also used a similar approach to investigate the effect of membrane roughness and lateral diffusion of receptors in mediating their interactions with ligands. Liu *et al.* [12,13] have developed a general computational framework that, in addition to explicitly representing the specific ligand–receptor interactions, also takes into account additional physiological factors such as the presence of glycocalyx barriers and shear forces due to blood flow. The internalization dynamics of NCs has also been explored in a number of recent studies [14,15].

Building on these theoretical developments that are faithful to the biophysical and thermodynamic factors, we propose to formulate a statistical mechanics-based model framework to predict the binding landscape of an NC in the native environment of a live endothelial cell in the vasculature, as well as to predict its tissue targeting behaviour across various organs in different species. We hypothesize that a comprehensive model for NC binding to live cells should account for a number of essential factors that include (i) the mechanical properties and protein expressions of the target cell membrane [10,16], (ii) surface functionalization of the NC [1,2,12,14,16], (iii) physiological and microenvironmental conditions such as blood flow, and margination due to the erythrocytes [2,16,17], (iv) specificity of receptor–ligand interaction [1,2,16,18], (v) non-specific interaction of the NCs in the target tissue [1,2] and (vi) receptor diffusion and translation and rotation of the NC [12,14]. In this sense, we present a next-generation framework that takes these various factors into account and predicts NC targeting to live cells in five different organs (lung, liver, heart, kidney and spleen) across two species (mouse and human).

**Figure 1.**
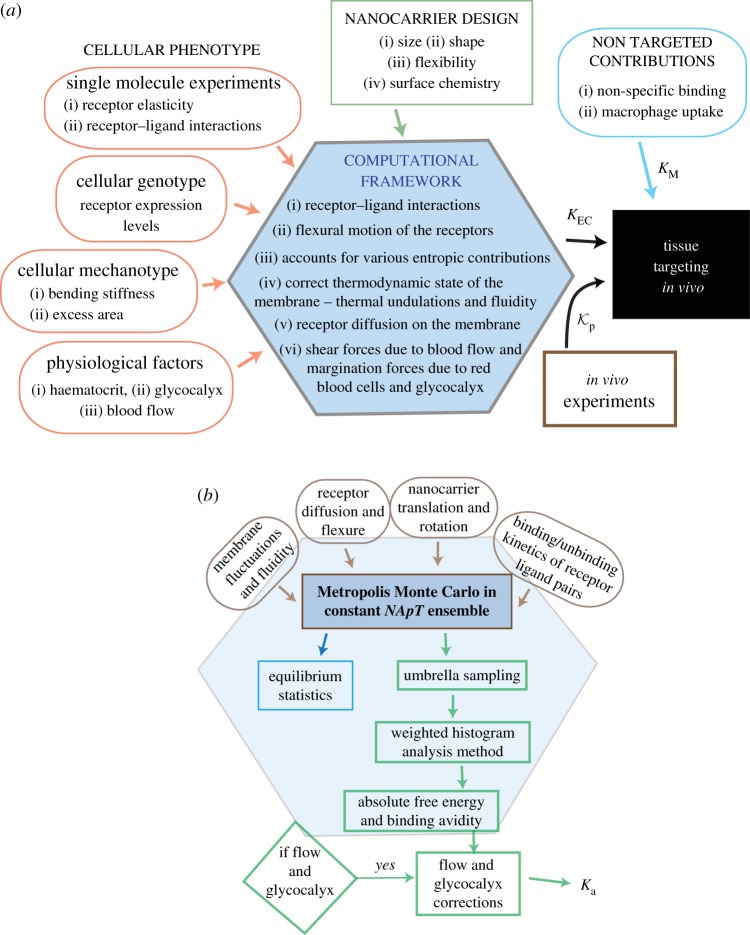
(*a*) A schematic of the proposed multiscale modelling framework to predict targeting of anti-intracellular adhesion molecule-1 (ICAM1; antibody) functionalized NCs across various organs in a given species. The input to the computational framework can be broadly classified into three categories that represent (i) cellular phenotype, (ii) NC design parameters and (iii) non-targeted contributions. $K_{p}$ denotes the partitioning coefficient measured in experiments. (*b*) A flow chart of the Metropolis Monte Carlo (MC)-based computational framework to compute the association constants (*K*_EC_ and *K*_M_) for NCs binding to live cells.

The binding dynamics of an NC is strongly influenced by the specificity of the receptor–ligand interactions, molecular stiffness such as the flexure of proteins, deformations of the cell membrane expressing the target receptors, expression levels of the target proteins, physiologically mediated external forces, such as forces due to blood flow and red blood cell margination, and entropic forces due to thermal undulations. The multiscale computational platform presented in this article is shown in figure 1*a* and details are presented in §2. It is based upon the framework of equilibrium statistical mechanics and couples continuum field models for cell membranes with coarse-grained molecular scale models for the NC, antibodies and target receptors. The information flow between the molecular and macroscopic degrees of freedom is defined in terms of energy functionals (integrals) and interaction potentials, that are described in detail in §2. The conformational state of a functionalized NC interacting with the cell membrane is evolved through a set of seven types of Monte Carlo moves (see §2), which on sufficient sampling yields the equilibrium conformations of the combined system and the required statistics on multivalent binding. The model parameters illustrated in figure 1*a*, as inputs to the computational framework, are self-consistently determined either from experimental measurements or using molecular simulations which makes this framework to be a *zero-fit* model. The main output of the MC simulation is the calculation of the free energy landscape for carrier binding to cells, which is quantified through umbrella sampling and the weighted histogram analysis method. This free energy landscape is used to compute the avidity of NC binding.

The illustration shown in figure 1*a* highlights the major components of the proposed computational platform. These components can be broadly classified as: (i) a set of input parameters for the coarse-grained and continuum models that completely define the protein expression and mechanical properties of the target cell membrane, the biochemical interactions of the receptor–ligand bond and the flexural rigidity of the target receptors, and experimentally controllable quantities such as the geometry and the surface chemistry of the functionalized NC; (ii) a computational engine based on Metropolis Monte Carlo techniques to exactly compute the association constant *K*_*a*_, for a specified mechano-chemical microenvironment and (iii) a framework that accounts for the targeted contributions due to NC binding to non-endothelial cells such as a macrophage. Most *in vivo* experiments only report the total concentration of NCs in the whole organ which also includes the binding and internalization of the carrier through a number of other non-specific pathways such as internalization through caveolin pits, and uptake by macrophages. Accurate representations of these non-specific contributions require a more detailed description of the tissue morphology and the physiological conditions. The modular design of our framework allows us to also include non-specific but highly relevant contributions such as physiological and microenvironmental conditions based on direct experimental measurements: these include non-specific capture mechanisms such as enhanced perfusion and oedema, capture through specific mechanisms not involving intracellular adhesion molecule-1 (ICAM1) (such as through the interaction of the antibody with FC receptors) [19], heterogeneity in tissue mechanics, and sub-cellular scale variations in the expression of the target proteins [2]. With these inputs from experiments, our model is able to predict the enhancement due to tissue targeting by quantitatively considering the biophysical factors outlined above. In the following sections, we describe the methodology and the procedure for parameter estimation before discussing the results.

## 2. Material and methods

### 2.1 Computational methods for nanocarrier binding to cell membrane

Monte Carlo protocols for the NC motion and adhesion to non-compliant surfaces have been extensively tested in previous works. Agrawal & Radhakrishnan [20] introduced the original version of the NC binding model using which they studied the effects of receptor flexure and the endothelial glycocalyx. Liu *et al.* [12] have extended this model by combining it with a powerful methodology for computing absolute binding free energies (described in the following subsection), through which they made successful comparisons to experiments probing NC binding to cells, tissue and NC unbinding in atomic force microscopy experiments. Later, Liu *et al.* [13] further extended their model to study the effects of shear flow and glycocalyx. Collectively, these works not only illustrate the model for NC binding in detail, but also explain how each parameter was obtained through independent well-controlled experiments, thereby obviating the need for any fitting of the data to obtain parameters. The values of some of the parameters determined from experiments (such as the flexural rigidity of the receptors) were also confirmed by independently carrying out molecular dynamics simulations [18]. The model presented in this work leverages the successes of the models described in the references cited above, and further extends these models by incorporating a crucial component, namely the compliance of the adhering interface.

The main components of the computational model are: (figure 1*b*) (i) the cell membrane, (ii) the position of the NC of radius *r*_NC_, (iii) the coarse-grained positions of the *N*_ab_ antibodies defined on the surface of the NC and (iv) the coarse-grained positions and flexure of the *N*_ant_ receptors defined on the curvilinear manifold defined by the membrane surface.

#### 2.1.1 Model for the cell membrane

The patch of the cell membrane to which the NC binds is represented as a continuum surface whose curvilinear area is denoted by A. The projection of the membrane surface onto the reference plane yields the projected area $A_{p}=L^{2}$. The excess area of the membrane which quantifies the difference between its curvilinear area A and the projected area $A_{p}$ is given by $A_{\mathrm{ex}}=100\times (A-A_{p})/A$.

For the purpose of numerical simulations, we represent the continuum membrane as a triangulated surface with *N*_m_ nodes, *T*_m_ triangles and *L*_m_ links, such that *N*_m_+*T*_m_−*L*_m_=0. The discretized form of the membrane elastic energy [21,22] for the triangulated surface may be given in terms of the principal curvatures as [23]:

2.1 $$H_{m}=\sum_{v=1}^{N_{m}}\frac{\kappa }{2}{\left(c_{1,v}+c_{2,v}\right)}^{2}A_{v}+\sigma A_{v}.$$

Here, *κ* and *σ* are, respectively, the effective bending rigidity and surface tension of the membrane of the target cell. *c*_1,*v*_ and *c*_2,*v*_ are the two principal curvatures at vertex *v* and $A_{v}$ denotes the curvilinear area of the discrete membrane associated with vertex *v*, such that the total curvilinear area is given by $A=\sum_{v=1}^{N_{m}}A_{v}$. We impose self-avoidance in the triangulated membrane by constraining the length of a link to be between *a*_0_ and $\sqrt{3}a_{0}$ [24], where *a*_0_ is the discretization length-scale, which is taken to be *a*_0_=10 nm.

#### 2.1.2 Model for the surface receptors

The receptor molecules are defined as cylindrical rods, with flexural angles *θ* and *ϕ* and length $L_{\mathrm{an}}$, on the surface of the membrane. Their base and tip positions are denoted by **a**_*b*_ and **a**_*t*_, respectively. When *θ*=*ϕ*=0, i.e. in the unflexed state, the orientation of a receptor $({\text{a}}_{t}-{\text{a}}_{b})/L_{\mathrm{an}}$ is along the normal to the membrane surface in its vicinity: all flexural angles are defined with respect to this direction and the length of the receptor is also defined with respect to the unflexed state such that $L_{\mathrm{an}}=|{\text{a}}_{t}-{\text{a}}_{b}|$. The flexural motion of a receptor *i*, with flexural rigidity *κ*_*f*_, is governed by the flexural energy:

2.2 $$H_{f}(\theta_{i})=\frac{\kappa_{f}}{2}\theta_{i}^{2}.$$

#### 2.1.3 Model for the ligands

The ligand molecules are defined as rigid rods of length $L_{\mathrm{ab}}$ such that their base positions **A**_*b*_ are on the surface of the NC and their tip positions **A**_*t*_ are defined according to some predefined orientations—in the case of a spherical NC the ligand molecules are defined along the radial direction. The binding interaction between a receptor *i* and a ligand *j* is modelled using the Bell potential:

2.3 $$H_{b}(d_{ij})=\left\{ \begin{array}{ll} H_{0}+\frac{1}{2}\kappa_{b}{\left(d_{ij}-d^{*}\right)}^{2} & \text{if }d_{ij}\leq d^{*}\\ 0 & \text{if }d_{ij}>d^{*}. \end{array}\right.$$

Here, *d*_*ij*_=|**A**_*t*,*i*_−**a**_*t*,*j*_| is the distance between the tip positions of the chosen receptor–ligand pair and d* is the range of the binding interaction. $H_{0}$ denotes the height of the energy barrier that separates the bonded and unbonded states and *κ*_*b*_ is the spring constant of a receptor–ligand bond.

The total energy of the NC–membrane system is given by

2.4 $$H_{t}=H_{m}+\sum_{i=1}^{N_{\mathrm{ant}}}H_{f}(\theta_{i})+\sum_{i=1}^{N_{\mathrm{ant}}}\sum_{j=1}^{N_{\mathrm{ab}}}H_{b}(d_{ij}).$$

#### 2.1.4 Monte Carlo moves

The conformational states of the system are evolved using a Metropolis Monte Carlo method that consists of seven independent moves: namely (1) a vertex move to simulate thermal fluctuations in the membrane, (2) a link flip to simulate membrane fluidity, (3) translation of the NC, (4) rotation of the NC, (5) diffusion of receptors, (6) receptor flexure move and (7) formation and breakage of receptor–ligand bonds. Moves (1–5) are performed according to the rules of canonical Monte Carlo while moves (6) and (7) are treated as rare events and are performed using the Rosenbluth sampling technique. Details of the various Monte Carlo moves are given in the electronic supplementary material, §S1. We note that with respect to the system (especially the membrane), we work in the ensemble of constant $\left(T,A_{p},\sigma \right)$ and hence the actual value of $A_{\mathrm{ex}}$ is determined by the system by fixing these variables [24]; here *T* is the system temperature.

### 2.2 Mean field model for cytoskeleton

The model for NC adhesion to the cell interface is expected to be influenced by the state of the cytoskeleton. While our model does not have an explicit representation for the cytoskeletal elements, the effects of the cytoskeleton are incorporated at the mean field level through the renormalization of the physical properties of the membrane, namely the values of *κ*, *σ* and $A_{\mathrm{ex}}$. In previous work, Qi *et al.* [25] have modelled a cytoskeletally fortified membrane using the Helfrich model by using a renormalized set of parameters described above for describing the mechanism of formation of the immunological synapse.

Several works in biomechanics have aimed to characterize cells based on mechanical measurements using a wide range of techniques such as flow and optical cytometry, manipulation using micropipette, optical tweezers and laser traps, and microfluidic devices (see [26–28] for comprehensive reviews). These studies have focused on whole-cell measurements. In the case of NC adhesion, the changes in mechanical properties are primarily caused by variations in the structure and organization of the cellular cytoskeleton [29] and the extracellular matrix [30]. Such sub-cellular scale rearrangements can significantly impact the mechanical properties of the cell membrane at length-scales smaller than cellular dimensions (i.e. tens of nanometres to less than 1 μm), a range which also corresponds to the scale at which the NC engages through multivalent interactions. The sub-cellular scale relevant to this discussion corresponds to the dimensions primarily set by the cortical cytoskeletal mesh, which has been estimated to be between *l*_*c*_=150–500 nm [31,32]. The mechanical properties of a patch of the cell membrane that spans the region between multiple cytoskeletal pinning points, with typical dimensions *l*_*c*_, can differ from the bulk because the nature of the thermal undulations (and the associated conformational entropy of the membrane) depends directly on *l*_*c*_. In addition, the total area of the membrane A, is in general, determined by the state of the cytoskeleton.

**Figure 2.**
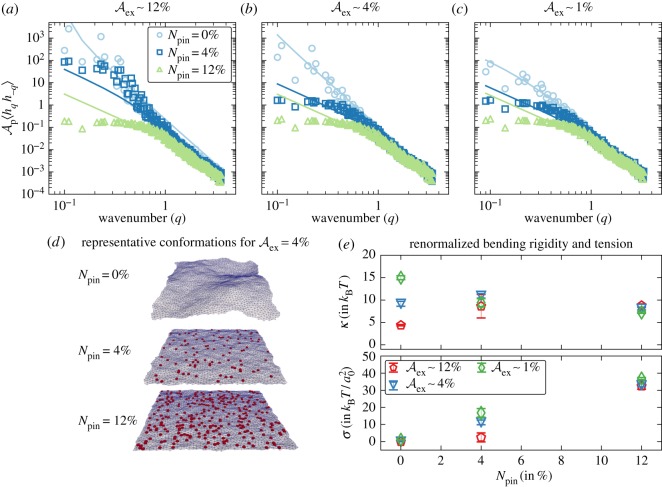
Fluctuation spectra of the height–height undulations on a membrane with different excess areas (*a*–*c*) shown for varying degrees of cytoskeletal pinning. (*d*) A depiction of membrane conformation and cytoskeletal pinning sites. (*e*) The renormalization of elastic parameters due to cytoskeletal pinning for different membrane excess areas. In panel (*e*), *a*_0_ denotes the length-scale for the triangulated membrane.

Drawing inspiration from the work of Qi *et al.* [25], we sought to describe the cytoskeletally fortified membrane using a renormalized set of parameters for the Helfrich model. We do so by introducing pinning sites on the membrane, which represent the binding of the cytoskeletal proteins to the membrane (figure 2). The pinning density is chosen to vary between 0 and 12% to mimic the distribution of *l*_*c*_ noted above [31,32]. We note that the lower end of the pinning density corresponds to *l*_*c*_>500 nm and the upper end corresponds to *l*_*c*_∼150 nm.

Even though the simulations are performed in curvilinear coordinates, for the sake of analysis of membrane undulations alone, we parametrize this surface in the Monge gauge and take the Cartesian *x*–*y* plane to be the reference surface—in this representation, every point on the membrane may be denoted by [*x*,*y*,*h*(*x*,*y*)] where *h*(*x*,*y*) is the height displacement along the *z*-direction. At non-zero temperatures, the spectrum of height undulations follow:

2.5 $$\langle h_{q}h_{-q}\rangle =\frac{k_{B}T}{A_{p}(\kappa q^{4}+\sigma q^{2})},$$

where *q* is the wavenumber, *k*_B_ the Boltzmann constant and *T* the absolute temperature, where *h*_*q*_ is the Fourier transform of *h*(*x*,*y*).

The results for the height–height undulation spectra (see equation (2.5)) for membranes with pinned sites are compared with those in the absence of pinning (figure 2). The results show that for all excess areas considered, the effect of cytoskeletal pinning is to renormalize the values of *κ* and *σ* and that the scaling in the spectra can be well described by those predicted by the Helfrich model with renormalized parameters. In particular, the pinning can alter the value of *κ* in either direction depending on the $A_{\mathrm{ex}}$, while its effect is to always increase the value of *σ*. Our results show that for length-scales that are comparable to *l*_c_, we can use the Helfrich model to investigate the binding free energy landscape for NC, and the renormalized parameters in figure 2 will enable us to consider effects of the cytoskeleton for a given state (represented by the pinning density and $A_{\mathrm{ex}}$). A detailed discussion on the methods to reliably estimate the properties of the cell membrane, namely, $\kappa ,\sigma ,A_{\mathrm{ex}}$, for any given cell type is given in §2.7.

### 2.3 Free energy analysis

#### 2.3.1 Umbrella sampling and weighted histogram analysis

The potential of mean force (PMF) for NC–membrane interactions is calculated using a macroscopic order parameter Δ*R*. This reaction coordinate is defined based on the combined conformational states of the NC and the membrane. If **R**_CM_ denotes the centre of mass of all membrane vertices within a distance of 2*r*_NC_ from the centre of the NC (denoted by **R**_NC_) then the macroscopic order parameter is defined as the distance Δ*R*=|**R**_CM_−**R**_NC_|. Umbrella sampling using a biasing potential **U**_bias_=*k*_bias_(Δ*R*−Δ*R*_0_)^2^/2 is performed at predefined values of Δ*R*_0_, with a window interval of *δR*=2 nm. The strength of the biasing spring is chosen to be *k*_bias_=2 *k*_B_*T*/(*δR*)^2^, i.e. the NC position can sample the entire window when it acquires 1 *k*_B_*T* of thermal energy. Each biased window is independently sampled for 900 million Monte Carlo steps (one half of the moves are distributed equally between moves (1–5) and other half are distributed between moves (6) and (7) in the ratio 2 : 5) and the histogram of Δ*R* is recorded every 10 MC steps. The histograms of Δ*R* from multiple windows are combined and unbiased using the Weighted Histogram Analysis method in order to compute the PMF denoted by W(ΔR). The PMF has a statistical significance since its Boltzmann weight exp⁡(−βW(ΔR)) is proportional to the probability to observe NC–membrane conformations with order parameter Δ*R*, and hence is a true measure of the stability of such configurations. Here *β*=(*k*_B_*T*)^−1^. In our studies, we have used the thermodynamic integration (TI) technique to sample configuration windows that are prone to endpoint catastrophe and determine the additive constant to be the relative free energy computed using TI [33].

We run our code on 32-cores in parallel, and each PMF calculation (this includes TI and WHAM) takes 32×48 CPU hours. Each PMF is run in quadruplate (for computing standard deviations), and in total four values of *κ*, four values of $A_{\mathrm{ex}}$, five values of *N*_ant_ and three *N*_ab_ densities were explored leading to an aggregate of 368 650 CPU hours of computing. The aggregate number of MC steps totalled 8640 trillion.

#### 2.3.2 Formulation of equations for binding avidity

In statistical mechanics, the binding avidity of a reaction is an exactly computable quantity and it has been applied with success in a number of problems involving small molecules and functionalized NCs [7,11,12,34,35]. Here, we present an equivalent form in terms of the PMF and the various entropic terms shown in figure 5 (details of the derivation are given in the electronic supplementary material, §S2). The association constant *K*_a_ for an NC functionalized with *N*_ab_/NC antibodies, forming *n*_*b*_ simultaneous bonds upon binding to a membrane surface expressing *N*_ant_ receptors is given by

2.6 $$\begin{array}{rl} K_{a} & =\underset{\begin{array}{c} \mathrm{combinatorial}\;\mathrm{entropy}\\ \mathrm{for}\;\mathrm{multivalent}\;\mathrm{binding} \end{array}}{\underbrace{\left(\frac{N_{\mathrm{ant}}!}{(N_{\mathrm{ant}}-n_{b})!\;n_{b}!}\right)\left(\frac{N_{\mathrm{ab}}}{n_{b}}\right)}}\underset{\begin{array}{c} \mathrm{NC}\;\mathrm{rotational}\\ \mathrm{entropy} \end{array}}{\underbrace{\left(\frac{\Delta \phi \Delta \theta \Delta \psi }{8\pi^{2}}\right)}}\\ & \;\times \underset{\begin{array}{c} \mathrm{translational}\\ \mathrm{entropy}\;\mathrm{of}\;\mathrm{receptors} \end{array}}{\underbrace{{\left(\frac{A_{R}^{b}}{A_{R}^{u}}\right)}^{n_{b}}}}\underset{\mathrm{enthalpic}\;\mathrm{contribution}}{\underbrace{\frac{A_{N}^{b}L_{z}}{\left(L_{z}-r^{*}\right)}\int_{0}^{r^{*}}dr\exp(-\beta W(r))}}. \end{array}$$

Here $A_{R}^{u}$ is the average area traversed by an unbound receptor molecule taken to be $A/N_{\mathrm{ant}}$, *L*_*z*_ is the dimension of the simulation box along the reaction coordinate, and r* denotes the cutoff length at which the conformational states of the NC and those of the membrane–receptor system cease to overlap, i.e. the NC can only exist in an unbound state.

### 2.4 Pharmacokinetic model for tissue targeting

Experimental measurements of NC targeting in an organ are typically expressed in units of ‘percentage injected dose per gram of tissue’ (denoted as %idg) and are performed at the tissue scale, though the binding interactions for commonly used NCs (sizes in the range of 50–250 nm) occur at the scale of a single cell.

The association constant, given in equation (2.6), can be used within the framework of PK models to determine the effective partitioning coefficient of the targeted NC within the tissue at steady state. This modified framework (described in detail in the electronic supplementary material, §S3) explicitly takes into account (i) the non-specific binding of NC represented by $K_{p}$, (ii) *K*_EC_, the association constant for the targeted adhesion of NCs to the endothelial cells and (iii) *K*_M_, the association constant for an NC adhering to a macrophage. The standardized uptake value for the NC, with injected concentration *C*_out_, in terms of $K_{p}$, *K*_EC_ and *K*_M_ is given by

2.7 $$\%\mathrm{idg}∼\frac{C_{\mathrm{tot}}}{C_{\mathrm{out}}}=\left\{K_{p}K_{\mathrm{EC}}C_{\mathrm{out}}+\frac{\varphi_{\mathrm{EC}}\;K_{\mathrm{EC}}\;L_{\mathrm{EC},b}}{D_{\mathrm{EC}}}\;C_{\mathrm{out}}\right\}\frac{L_{\mathrm{cap}}}{L_{\mathrm{EC},b}}+\frac{\varphi_{M}\;K_{M}\;L_{M,b}}{D_{M}}\;C_{\mathrm{out}}\times \frac{L_{\mathrm{cap}}}{L_{M,b}},$$

Here, *ϕ*_*X*_ and *D*_*X*_ denote the concentration and diameter of endothelial cells (*X*=*EC*) and other cells (*X*=*M*) in the target tissue. The variable $L_{X,b}$ is the value of r* defined in equation (2.6) for the different cell types and the variable *L*_cap_ represents the size of the cell free layer in the capillary in which the NC is perfused.

We present all %idg scores in scaled units given by *η*=(%*idg*)_org,sp_/(%*idg*)_lung,sp_, where the subscripts ‘org’ and ‘sp’ represent the target organ and species, respectively. The corresponding predictions from the computational model and experiments are denoted by *η*_sim_ and *η*_exp_, respectively. We note that in these ratios the values of *L*_cap_ and *C*_out_ do not feature, while all other parameters are summarized in §2.7. We predict the endothelial targeting of anti-ICAM1-coated NCs in five different organs in mouse and compare them to targeting levels measured in *in vivo* experiments. The error bars in *η*_sim_ denote the standard deviation in the %idg scores, normalized by the %idg score for the lung. This standard deviation is determined by computing %idg scores for *K*_EC_−Δ*K*_EC_, *K*_EC_, *K*_EC_+Δ*K*_EC_, where *K*_EC_ denotes the mean value of the association constant and Δ*K*_EC_ its standard deviation.

In our model, we note that the factor $K_{p}$ does include non-specific uptake including via internalization mechanisms such as phagocytosis, macrophage capture, etc. The targeting will enhance such uptake mechanisms because the concentration of NC on the EC surface is enhanced due to targeting. This effect is included in our model formulation. However, the ICAM-mediated internalization is not included here. *In vivo* measurements (described in the next section) are performed in a timescale where the NC internalization will be minimal (as explained in that section). This justifies our neglect of NC internalization in the current formulation. We do include the fraction of NCs captured on the EC in the %idg calculations outlined above, which implies that NC fraction captured on the EC are accounted for in the tissue targeting estimates.

We further note that, for longer times, the effect of internalization can be included by augmenting the $K_{p}$ values of ICAM1-mediated NC over and above the values for those for IgG-coated NCs. We note that in the future we will investigate the specific ICAM1-coated uptake by extending our model to study this process directly.

### 2.5 *In vivo* targeting to vascular endothelium in mice

Anaesthetized C57BL/6 female mice (16–24 g, Harlan) were injected intravenously via jugular vein with NCs coated with murine anti-ICAM1 (YN1 clone, Biolegend) or control rat IgG (Jackson Labs). The injected dose was approximately 200 μl (or approx. 10 mg kg^−1^) with a tracer amount of antibody-coated ^125^I-labelled NC. Blood was collected from the retro-orbital plexus at 30 min post-injection and organs (heart, kidneys, liver, spleen and lungs) were collected at 30 min post-injection. Radioactivity and weight of the samples were determined to calculate NC targeting. These studies were carried out in accordance with the Guide for the Care and Use of Laboratory Animals as adopted and promulgated by the US National Institutes of Health. For a direct comparison, we quantified endothelial targeting as a function of organ uptake of NCs in mice. Full coverage of NCs was expressed as 100% endothelium targeting. We note that in the time of 30 min post-injection, the effect of ICAM1-mediated internalization of NC to tissue should be minimal because the timescale for such an internalization is about hour [36,37].

### 2.6 Comparison between model and *in vivo* experiment

We compare the %idg values computed from our model under various scenarios to those measured in experiments. In order to assess the predictive accuracy of our model, we compute the Pearson's correlation coefficients [38] (denoted by *r*^2^) between model and experiment. The calculation of *r*^2^ considers both mean as well as the standard deviation in the computed and experimental values. We do so through a bootstrapping procedure, where five sets of 2000 datapoints are generated from a Gaussian distribution based on the mean and standard deviation of the calculated or experimental data. For each set of 2000 points, the *r*^2^ value is computed between model and experiment. We report the mean *r*^2^ over all 10 000 data points and a standard deviation in *r*^2^ based on the *r*^2^ values from each of the five sets.

The significance value (*p*-value) for the *r*^2^ for each model prediction is established by comparing the *r*^2^ for our tissue targeting model with that from a model representing the null hypothesis corresponding to no targeting. Specifically, the model corresponding to the null hypothesis assumes that the %idg is given by just $K_{p}$. The *p*-value is computed using an unpaired *t*-test between our model and the model for the null hypothesis.

### 2.7 Parameter estimation

While our earlier works have discussed the methodologies for estimating model parameters [12,13,39], the detailed parameter sets used in our current model and the corresponding references are summarized for completeness in table 1.

**Table 1.** Parameters used in the model.

parameter	value	ref.
simulation surface area	0.25 μm^2^	
simulation height	0.5 μm	
NC diameter (2*r*_NC_)	100 nm	[40]
receptor length $\left(L_{\mathrm{an}}\right)$	19 nm	[41]
antibody length $\left(L_{\mathrm{ab}}\right)$	15 nm	[42]
number of receptors (*N*_ant_)	2000	[40]
number of antibodies per NC (*N*_ab_)	12–162	[40]
free energy change at equilibrium per bond $\left(H_{0}\right)$	−7.98×10^−20^ J	[40]
bond spring constant (*κ*_b_)	1 N m^−1^	[43,44]
receptor flexural rigidity (*κ*_f_)	7000 pN nm^2^	[45]
flow shear rate (*S*)	6000 s^−1^	[46]
glycocalyx height (*h*_glyx_)	100 nm	[45]
glycacalyx stiffness (*k*_glyx_)	3.9×10^9^ J m^−4^	[47]
system temperature (*T*)	300 K	
*D*_EC_	5 μm	[48,49]
*D*_M_	5 μm	[48,49]
$L_{\mathrm{EC},b}$	100 nm	r* from figure 4
$L_{M,b}$	100 nm	r* from figure 4

#### 2.7.1 Estimating model parameters for ICAM1-targeted nanocarriers in mouse

In order to make reliable predictions for an NC binding to the target tissue, it is essential to accurately estimate the model parameters given in equation (2.7). This involves two set of measurements: (i) estimation of (*κ*, $A_{\mathrm{ex}}$ and *N*_ant_)_*X*_ to compute *K*_*X*_ for *X*=*EC* and M, and (ii) estimates for $K_{p}$, *ϕ*_*X*_, $L_{X,b}$ and *D*_*X*_ to compute the biodistribution.

#### 2.7.2 Estimating protein expression levels

**Figure 3.**
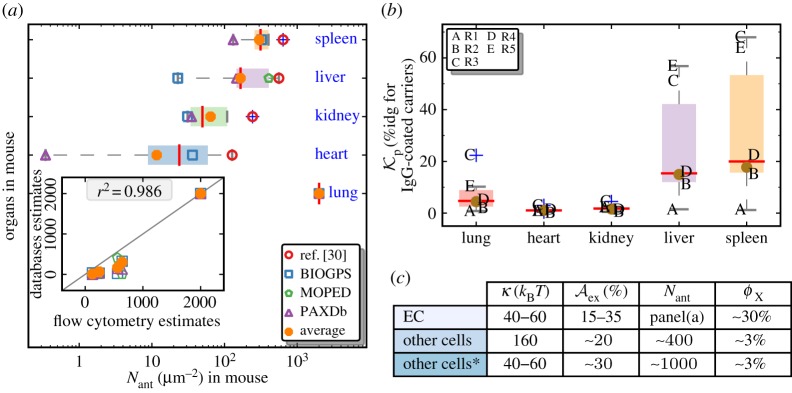
(*a*) ICAM1 expression levels in the lung, liver, heart, kidney and spleen of mouse, determined using data from radio-labelling experiments [50], mRNA measurements from BIOGPS [51] and proteome mass spectrometry from PAXDb [52]. The protein levels are expressed in units of number per square micrometre of the cell surface. The inset shows the correlations in the expression levels determined using radio-labelling experiments and other high-throughput methods. (*b*) The partitioning coefficient $K_{p}$ reported for IgG-coated NCs in five different organs in mouse (references R1–R5) R1: [53], R2: [54], R3: [40], R4: [55], R5: [56]. (*c*) The mechanical parameters and protein expression levels in endothelial cells (EC), other cells, (here the * represents the activated form of these cells). The corresponding concentration in the tissue is also shown alongside.

The expression levels of ICAM1 molecules (*N*_ant_) for five major organs in mouse—namely the lung, liver, kidney, heart and spleen—are estimated by combining data from *in vitro* flow cytometry measurements [50], *in vivo* radio labelling measurements [50], mRNA expression levels reported in the BIOGPS database [51] and mass spectrometry measurements of proteome levels reported in the MOPED and PAXDb [52] databases. The number of ICAM1 molecules per square micrometre of the cell surface is shown in the main plot of figure 3*a*, and our results show that *N*_ant_ varies between 2 and 3 orders of magnitude across the various organs, with the expression being largest in the lungs (approx. 2000 ICAM1 μ*m*^−2^) and smallest in the heart (approx. 50 ICAM1 μ*m*^−2^), and the former is consistent with previous estimates for ICAM1 in mouse lungs [2]. The values of *N*_ant_ estimated from mRNA and whole-proteome measurements correlate very well with those measured using flow cytometry experiments, and this is shown in the inset to figure 3*a*. We note that while the correlation is good (with a reported *r*^2^=0.98), the direct measures of accessible protein levels are the best choice for our model, where such data are available. Furthermore, we have also determined *N*_ant_ for other cells (e.g. resting monocytes and activated macrophages) using data from the BIOGPS database, and the corresponding values are given in figure 3*c*. Later in §3, we compute NC adhesion for the range of ICAM densities, *N*_ant_=200, 500, 1000, 2000 and 4000 ICAM1 μ*m*^−2^. We note that these densities are based on the projected area $A_{p}$ rather than the total area A so that *N*_ant_ and $A_{\mathrm{ex}}$ can be independently varied in our analyses.

#### 2.7.3 Estimating mechanical properties of the target cell membrane

The bending rigidity and interfacial tension for cells are obtained from the work of Pontes *et al.* [57]. Based on our recent work [58], we have used a tether pulling assay to investigate the sub-cellular scale excess areas in different types of cells. Our estimates showed the bending rigidity and the excess area for most endothelial cells to be in the range of 40–60 *k*_B_*T* and 10–30%, respectively. On the other hand, the bending rigidity of resting and activated macrophages varied substantially with *κ*=160 *k*_B_*T* and *κ*=40 *k*_B_*T*, respectively, while the excess areas for both these classes of macrophages were found to be around 20%.

The parameters for *κ* and *σ* obtained from the experiments noted above are already renormalized parameters including the effect of the cytoskeletal effects and can therefore be directly used in our Helfrich model. The length-scale of the membrane undulations probed in our model is set by the NC size which closely aligns with that explored experimentally by Pontes *et al.* [57] from which the representative values of the bending rigidity and membrane tension for the cell membrane patch are derived. Moreover, the $A_{\mathrm{ex}}$ of the membrane is interpreted to be the membrane area stored in membrane folds and ruffles. The role of thermal fluctuations in our simulations is to realize that the membrane relaxations in folded membranes (i.e. large $A_{\mathrm{ex}}$) are different from those devoid of folds (i.e. small $A_{\mathrm{ex}}$).

#### 2.7.4 Physiology-dependent parameters

The non-targeted partitioning coefficient $K_{p}$ (see equation (2.7)) is another important input parameter that is gleaned from experimental data. We take $K_{p}$ to be the biodistribution of Immunoglobulin (IgG) antibody-coated NCs, which is commonly used as a control in experiments and is believed to be an effective measure of NC targeting through all possible non-specific mechanisms. Figure 3*b* shows the values of $K_{p}$ from six different experiments [40,53–56,59] that have investigated the targeting of 100–200 nm anti-ICAM1-coated NCs to various organs in mouse. The measured levels of IgG carriers are consistently found to be larger in the liver and spleen which are known to be the primary clearance organs. The concentration of endothelial cells (*ϕ*_EC_) and of macrophages (*ϕ*_M_) in the target tissue varies across different organs, with the latter being high in clearance organs such as the liver and spleen and negligible in organs such as the lung and heart. Based on previously reported values [60], we set *ϕ*_EC_=30% for all organs, and *ϕ*_M_=3% for spleen and liver and 0% for the rest.

#### 2.7.5 Parameters for nanocarrier characterization

In table 2, we present the simulation parameters for NC characterization obtained from various sources in the literature.

**Table 2.** Parameters for nanocarrier characterization.

experiment	NC radius (nm)	*N*_ab_
Kolhar *et al.* [53]	100	162
Ferrer *et al.* [54]	50	100
Muro *et al.* [40]	100	162
Papedemetriou *et al.* [55]	115	162
Hsu *et al.* [56]	50	100
experiment (41)	50	41
experiment (100)	50	100
experiment (162)	50	162

#### 2.7.6 Interpolating the association constant *K*_a_

An important question that arises in post-processing is how to estimate the association constant for arbitrary values of the receptor density, while we only have data for 200, 500, 1000, 2000 and 4000 ICAM1 μ*m*^−2^.

A strategy we have adopted is to linearly interpolate the total free energy F which is related to the association constant $K_{a}\propto \exp(-\beta F)$, where *β*=(*k*_B_*T*)^−1^. Consider two receptor densities *n*_1_ and *n*_2_ with corresponding free energies $F_{1}$ and $F_{2}$, and association constants *K*_a1_ and *K*_a2_. The free energy for an intermediate concentration n* can be linearly interpolated to be

2.8 $$F^{*}=F_{1}+(F_{2}-F_{1})\frac{n^{*}-n_{1}}{n_{2}-n_{1}}.$$

The association constant *K*_a*_ then takes the form:

2.9 $$\begin{array}{rl} K_{a*} & =\exp(-\beta F^{*})∼\exp\left(-\beta \left(F_{1}+(F_{2}-F_{1})\frac{n^{*}-n_{1}}{n_{2}-n_{1}}\right)\right)\\ & =\exp(-\beta F_{1})\exp\left(-\beta \frac{n^{*}-n_{1}}{n_{2}-n_{1}}F_{2}\right)\exp\left(\beta \frac{n^{*}-n_{1}}{n_{2}-n_{1}}F_{1}\right)\\ & =\exp\left(-\beta \frac{n_{2}-n^{*}}{n_{2}-n_{1}}F_{1}\right)\exp\left(-\beta \frac{n^{*}-n_{1}}{n_{2}-n_{1}}F_{2}\right)\\ & ={\left(K_{a1}\right)}^{\left(n_{2}-n^{*})/(n_{2}-n_{1}\right)}{\left(K_{a2}\right)}^{\left(n^{*}-n_{1})/(n_{2}-n_{1}\right)}. \end{array}$$

## 3. Results and discussions

### 3.1 Can mechanical properties of the cell membrane be important determinants of nanocarrier avidity?

We hypothesize that (i) variations in the mechanical properties (*elastic parameters*) of the target cell membrane (the bending stiffness, *κ*, and the membrane excess area, $A_{\mathrm{ex}}$), (ii) variations in the expression levels of the target receptors (*N*_ant_) and (iii) the expression level of the affinity ligand on the NC (*N*_ab_/NC) are the key determinants of NC avidity. We have tested these hypotheses by systematically varying *N*_ab_/NC, *N*_ant_, *κ* and $A_{\mathrm{ex}}$ in our model. In order to compare with our experimental data for the endothelial targeting of anti-ICAM1-coated NC, we choose a 100 nm spherical NC in the model together with the receptor–ligand interactions that mimic the interaction of a specific anti-ICAM1 molecule with the ICAM1 receptor. Details of the parameters and methods to estimate them from experimental data can be found in §2.7.

**Figure 4.**
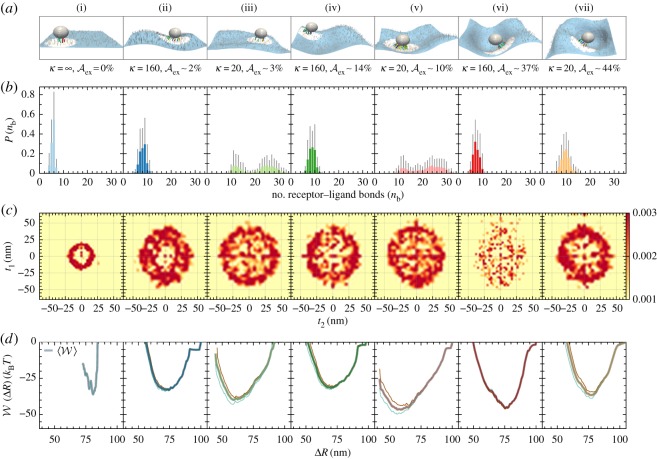
(*a*) Snapshots of an NC, with *N*_ab_=162/NC, bound to seven characteristically distinct cell membrane types—(i) κ=∞, $A_{\mathrm{ex}}=0\%$, (ii) *κ*=160 *k*_B_*T*, $A_{\mathrm{ex}}∼2\%$, (iii) *κ*=20 *k*_B_*T*, $A_{\mathrm{ex}}∼3\%$, (iv) *κ*=160 *k*_B_*T*, $A_{\mathrm{ex}}∼14\%$, (v) *κ*=20 *k*_B_*T*, $A_{\mathrm{ex}}∼10\%$, (vi) *κ*=160 *k*_B_*T*, $A_{\mathrm{ex}}∼37\%$ and (vii) *κ*=20 *k*_*b*_*T*, $A_{\mathrm{ex}}∼44\%$—through specific, multivalent receptor–ligand interactions (the corresponding movies can be found in the electronic supplementary material, §S4, Movies M1–M7). The solid lines and dots on the membrane surface denote bonded and unbonded receptors, respectively. (*b*) The equilibrium distribution of the number of simultaneous receptor–ligand bonds. (*c*) The colour map showing the spatial localization of the bound receptors with respect to the centre of the NC. (*d*) The PMF computed as a function of the separation of the NC from the membrane, parametrized by the collective order parameter Δ*R*, see §2. Each panel shows four different PMF curves which are quadruplets of simulations based on which we compute the standard deviation. The shaded line represents the mean PMF computed by aggregating the four PMF curves.

The equilibrium macroscopic conformations of a cell membrane are primarily governed by the two elastic parameters *κ* and $A_{\mathrm{ex}}$. For cells, the value of *κ* varies between 20 and 200 *k*_B_*T* [24] and the membrane excess area may vary from 0 to 300% [61,62], depending on the lipid composition and the degree of cytoskeletal pinning. In an earlier study, we have estimated this range to be between 1 and 35% [58] depending on the cell type. We note that the planar substrates used in earlier NC adhesion models characterize membranes with κ=∞ and $A_{\mathrm{ex}}\%=0$. We, here, hypothesize that variations in *κ* and $A_{\mathrm{ex}}$ strongly influence the equilibrium conformations and fluctuations of the cell membranes, and these in turn can alter the effective free energy landscape for NC binding thereby directly influencing carrier avidity. Figure 4*a* shows an anti-ICAM1-coated NC, with *N*_ab_=162/NC, bound to cell membranes with varying values of *κ* and $A_{\mathrm{ex}}$. We focus on the interaction of the NC with flexible (*κ*=20 *k*_B_*T*) and stiff (*κ*=160 *k*_B_*T*) membranes, with excess areas that fall into three distinct regimes: (1) small ($A_{\mathrm{ex}}\%=0$–5), shown in panels (ii) and (iii), (2) intermediate ($A_{\mathrm{ex}}\%=10$–20), shown in panels (iv) and (v), and (3) large $\left(A_{\mathrm{ex}}>35\%\right)$, shown in panels (vi) and (vii). We also compare the binding statistics to those observed for NCs interacting with a flat substrate that is shown in panel (i) of figure 4. Since all the membrane conformations have a fixed projected area $L^{2}$ (here L=510 nm), a membrane with non-zero values of $A_{\mathrm{ex}}$ accommodates the additional area through undulations. We now test our hypothesis in three steps: (a) by examining the statistics of multivalent interactions; (b) through determining the spatial map of the bound receptors, which have been previously identified to be markers of NC avidity [12] and (c) by directly computing the adhesion free energy landscape. In all cases, we compare these measures to that obtained for a planar membrane with similar values of *N*_ant_ (figure 4).

### 3.2 Multivalency of nanocarrier binding is a strong function of the mechanical variables of the binding surface

Multivalency, or the number of simultaneous (anti-ICAM1)–ICAM1 bonds formed between the ligands on the NC and the receptors expressed on the cell membrane is an important marker of NC avidity—it is a direct measure of the enthalpic contributions to the binding process. Figure 4*b* shows *P*(*n*_b_), the probability distribution of the multivalency *n*_b_, computed at equilibrium for an anti-ICAM1 functionalized NC in contact with each of the seven classes of cell membranes considered earlier; here *N*_ab_=162/NC and *N*_ant_=2000 ICAM1 μm^−2^. The number of multivalent bonds formed by the NC is highly sensitive to variations in both *κ* and $A_{\mathrm{ex}}$, and it may be seen that the stiffer membranes consistently stabilize 5–10 multivalent bonds for all values of $A_{\mathrm{ex}}$, while flexible membranes with similar excess areas stabilize between 10 and 30 bonds in the small and intermediate $A_{\mathrm{ex}}$ regimes and between 5 and 15 multivalent bonds in the large $A_{\mathrm{ex}}$ regime. Further, there is a sharp difference in the distribution profile *P*(*n*_b_), which for stiff membranes is normally distributed around a peak value of *n*_b_≈8, for all the three cases of $A_{\mathrm{ex}}$, while that for flexible membranes has a significantly broad and bimodal distribution in the small and intermediate regimes and crosses over to a normal distribution, with a peak at *n*_b_≈10, for large values of $A_{\mathrm{ex}}$. *P*(*n*_b_) for the membrane in the small deformation limit (panel (ii)) is consistent with that seen for flat substrates (panel (i)) and also agrees with that reported in earlier studies using planar substrates [12,13]. These results validate the hypothesis that *κ* and $A_{\mathrm{ex}}$ of the target cell membrane can significantly alter the multivalency for the adhesion of functionalized NCs. However, these findings do not by themselves establish if the observed enhancement in NC avidity is solely due to the increase in enthalpy (due to the shift in the distribution and peak value of *n*_b_) or if the entropic terms play a significant role as well. For example, it has been previously shown by us that in the case of flat substrates, the loss in the translational entropy of the diffusing receptors competes against the enthalpic gain due to receptor–ligand binding, and the interplay between these contributions limits the peak value of *n*_b_≈3. In the following, we explore how such an effect manifests for cells with different membrane properties.

### 3.3 Bound receptors show different localization patterns under varying mechanical properties of the binding surface

The area traversed by a bound ICAM1 receptor on the cell surface is an indicator of the loss of translational entropy of the receptors on a cell surface due to multivalent engagement of receptor–ligand bonds [12]. Our results in figure 4*b* show the spatial map *P*(*t*_1_,*t*_2_) to find a bound receptor–ligand pair at a point [*t*_1_,*t*_2_] on a tangent plane defined by the mean orientation of the bound receptors (see the electronic supplementary material, §S2)—here the point [0,0] denotes the projection of the NC's centre of mass onto this tangent plane. It may be seen from figure 4*b*,*c* that: (1) receptors on a flat substrate that are bound to the NC are localized to an annulus-like region, consistent with that seen in earlier studies [12] and (2) in the presence of thermal undulations of the membrane (which is absent in the flat substrate model), *P*(*t*_1_,*t*_2_) shows a larger spread and a diffuse pattern pointing to how bending modes in the membrane can manifest as additional binding modes for receptors on its surface. Our results, which display widely varying spatial maps of receptor–ligand pairs for different contexts of the cell membrane, clearly indicate that the translational entropy contribution can be cell-type (context) specific. Other enthalpic and entropic terms can also become significant in determining NC adhesion and these are discussed in the following.

### 3.4 Potential of mean force, which determines the enthalpic as well as entropic contributions to the binding avidity, is a strong function of the mechanical properties of the binding surface

The PMF W(ΔR) is characteristic of the effective free energy landscape for NC binding and hence is an important contributor to the binding avidity of the NC (see §2 for details of PMF calculation). We fix its absolute free energy levels using a bridging technique based on thermodynamic integration [33], such that in all cases the unbound state has zero free energy. The PMF for all seven types of cell membranes is shown in figure 4*d*, and we find that both the form and the minimum value of the PMF ($W_{\min}$) are strong functions of *κ* and $A_{\mathrm{ex}}$, an observation consistent with our hypothesis. It should be noted that the free energy landscape for NCs adhering to cell membranes is smoother and also broader compared with that seen for flat substrates. In the case of flexible membranes, $W_{\min}$ is the lowest for membranes with intermediate excess areas $\left(10\%<A_{\mathrm{ex}}<20\%\right)$ pointing to a synergy between the characteristic undulations in this regime and NC adhesion. It is interesting to note that flexible membranes with very large excess area (see panel (vii)) do not promote NC adhesion and effectively behave like a flat substrate. This is primarily due to the mismatch between the characteristic wavelengths of membrane undulation and the wavelength defined by the particle size.

### 3.5 Expression levels of target proteins differentially affect nanocarrier targeting under varying mechanical properties of the binding surface

**Figure 5.**
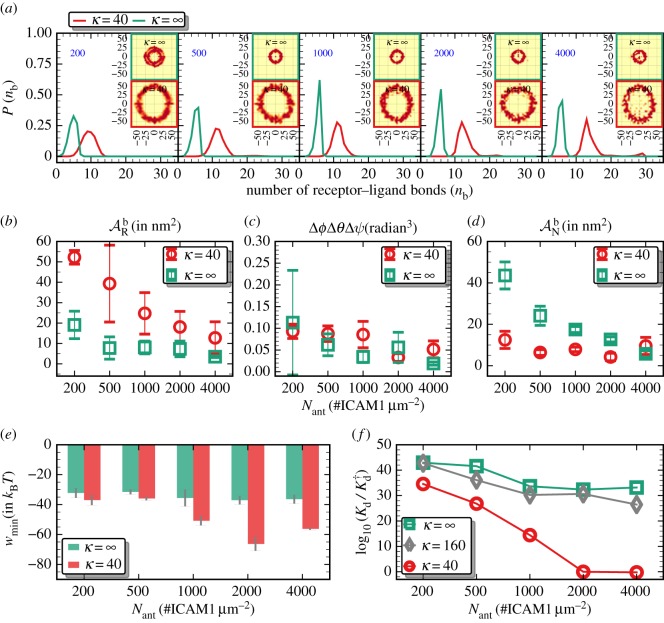
Statistics of NC, with *N*_ab_=162/NC, binding to a cell membrane for five different receptor densities *N*_ant_=200, 500, 1000, 2000 and 4000 ICAM1 μ*m*^−2^. (*a*) The probability density of multivalent interactions *P*(*n*_b_) for flat substrates (κ=∞) and for undulating membranes with *κ*=40 *k*_B_*T* and $A_{\mathrm{ex}}=10\%$. The insets in the top panel show the spatial localization of the bound receptors. (*b*) The average area traversed by a bound receptors molecule, (*c*) the rotational volume accessible to an NC in its bound state, (*d*) the area traversed by the NC in its bound state, (*e*) the minimum value of the PMF and (*f*) the dissociation constant *K*_d_ (in pM) as a function of *N*_ant_, for membranes with κ=∞, 160 and 40 *k*_B_*T*.

**Figure 6.**
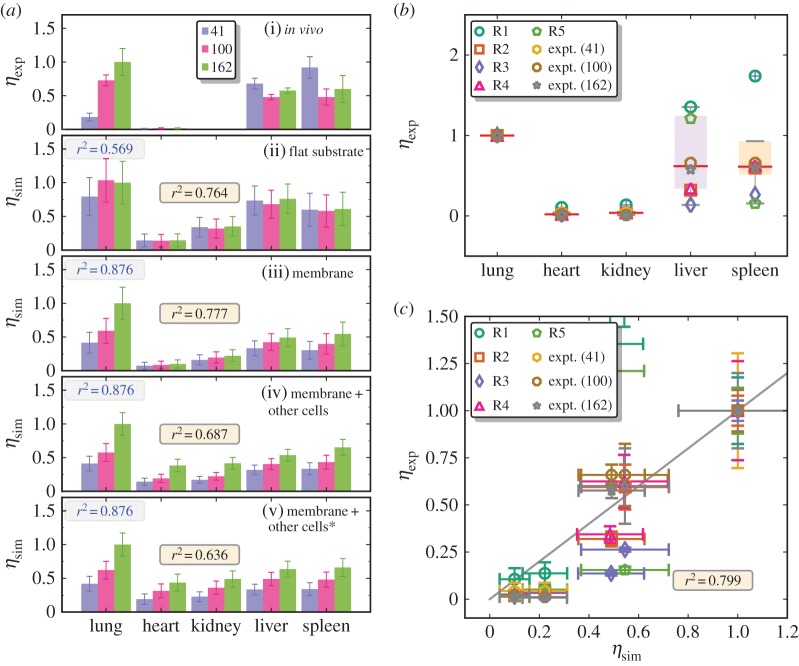
(*a*) Experimentally measured (panel (i)) and computationally predicted (panels (ii–v)) tissue targeting of anti-ICAM1-coated NCs, with *N*_ab_=41, 100 and 162/NC, in five different organs in mouse. The correlation coefficient computed across all organs is shown in the centre of each panel while that computed only for the lung is shown in the top left. (* indicates the activated form of cells.) (*b*) Normalized uptake levels of ICAM1 targeted NCs across five different organsin mouse from six independent experiments [40,53–56]. (*c*) A comparison of the experimental tissue targeting to the corresponding predictions of the computational model. The solid line in (*c*) represents a linear correlation and error bars in all panels represent two standard deviations.

The density or the expression level of the target ICAM1 molecules is an obvious major factor that determines the binding affinity of the NC [2]. Modulations in ICAM1 density not only modulate the binding specificity of the NC, but also change its selectivity to the target cell [63]. We hypothesize that the effect of ICAM1 expression on NC binding will depend on the mechanical properties of the binding surface. In order to test this hypothesis, we systematically examined the various measures that quantify the binding characteristics of an anti-ICAM1-coated NC interacting with a cell membrane (with *κ*=40 *k*_B_*T* and $A_{\mathrm{ex}}=10\%$) by varying the receptor density (figure 5). We have chosen five values, *N*_ant_=200, 500, 1000, 2000 and 4000 ICAM1 μ*m*^−2^, that are representative of the lung endothelium of a mouse with under-expressed, normal and over-expressed ICAM1 levels. In figure 5, as before, we quantify the various contributions to NC avidity using a set of measures, namely (1) *P*(*n*_b_) (figure 5*a*), (2) $A_{R}^{b}$, the average area traversed by a receptor molecule in its bound state (figure 5*b*), (3) Δ*ϕ*Δ*θ*Δ*ψ*, the rotational volume accessible to the NC in its bound state (figure 5*c*), (4) $A_{N}^{b}$, the average area traversed by the NC when bound to the membrane surface (figure 5*d*), (5) $W_{\min}$ (figure 5*e*) and (6) the dissociation constant $K_{d}=K_{a}^{-1}$ (figure 5*f*); *K*_a_ is defined in equation (2.6), which encodes the exact relation between these observables and the binding avidity.

From the main panels in figure 5*a*, it is evident that, unlike for flat substrates, *P*(*n*_b_) for a cell membrane shows a peak at higher multivalency with a broader distribution. Both the flat substrates and the cell membranes show a distinct annulus-like pattern with the annulus size consistent with the respective multivalency distributions; this is shown in the insets to figure 5*a*. Since *P*(*t*_1_,*t*_2_) is related to the lateral organization of the bound receptors, it is a determinant of the entropic contribution due to the receptors. This contribution is depicted in figure 5*b*: here $A_{R}^{b}$ is the average area traversed by a bound receptor, and it is evident that the bound receptors are highly mobile (large areas) at low receptor densities and more localized (small areas) at larger receptor densities. This is a direct measure of the translational entropy of the receptors, and points to the fact that the entropy loss in the receptor degrees of freedom increases with increasing values of *n*_b_. We also find that the values of $A_{R}^{b}$ for flat substrates, which are always smaller than those for cell membranes, follow a similar behaviour as a function of receptor density.

In a similar manner, the rotational entropy may be estimated from the rotational volume Δ*ϕ*Δ*θ*Δ*ψ*, where Δ denotes the standard deviation in the three Euler angles *ϕ*, *θ* and *ψ* characterizing NC orientation about its centre of mass (see §2). The accessible rotational volume as a function of the receptor density is given in figure 5*c*, and we find that its estimate for both systems (cell membrane and flat substrate) are not sensitive to variations in *N*_ant_.

We have also computed $A_{N}^{b}$, the average area traversed by the centre of mass of the NC in its bound state, which is a good estimate of its translational entropy. As is shown in figure 5*d*, the value of $A_{N}^{b}$ for NCs bound to cell membranes is a weakly varying function of *N*_ant_ which, as explained before, is due to the high affinity of the NC for the cell membrane even at low receptor densities. On the other hand, $A_{N}^{b}$ for flat substrates shows a monotonic decrease with increase in *N*_ant_, which is reflective of a monotonic increase in the multivalency of the NC that is solely governed by the receptor expression levels (figure 5*a*).

Figure 5*e* shows the depth of the PMF $\left(W_{\min}\right)$, which is the free energy difference between the unbound and the equilibrium bound state of an NC. It is noted that $W_{\min}$ for a flat substrate is unaltered by changes in *N*_ant_, while its response in the case of cell membranes is non-monotonic. At low and intermediate surface densities, as is shown for membranes expressing 200–2000 ICAM1 molecules μ*m*^−2^, $W_{\min}$ decreases with increase in *N*_ant_, with the depth being maximum for *N*_ant_=2000, and increases to a higher value when *N*_ant_=4000. This behaviour indicates that a functionalized NC does not show enhancement in binding free energy if the target receptor expression exceeds a critical threshold value.

The above observations validate our hypothesis that the avidity of a functionalized NC is a result of the complex interplay between the various energetic and entropic contributions in the system. This is further exemplified in figure 5*f* where we depict the effect of *N*_ant_ and *κ* on the dissociation constant *K*_d_, computed from the association constant *K*_a_ as *K*_d_=(*K*_a_)^−1^; for a definition of *K*_a_, see equation (2.6). The values are reported as ratios normalized by *K*^†^_d_, which is the value of *K*_d_ for a system with *κ*=40 *k*_B_*T*, $A_{\mathrm{ex}}=10\%$ and ICAM1 expression=2000 molecules μ*m*^−2^, representative values for regular EC in lung. It is evident from figure 5*f* that the computed values of *K*_d_ are strongly influenced by *κ* and ICAM1 expression.

### 3.6 Predicting the endothelial targeting of ICAM1-coated nanocarrier in mouse

Figure 6*a*(i) shows the *in vivo* endothelial targeting of ICAM1-targeted NCs, with *N*_ab_=41, 100 and 162/NC, in the lung, heart, kidney, liver and spleen of mouse [12]. These results show that a high level of targeting is achieved in the lung as evidenced by the increase in *η*_exp_ with increasing *N*_ab_. On the contrary, the uptake in liver and spleen is non-targeted since *η*_exp_ is not sensitive to increase in *N*_ab_. In panels (ii)–(v) of figure 6*a*, we present the corresponding computational predictions using four different model scenarios (see equation (2.7)) to quantify tissue targeting: (1) to flat substrates, (2) to endothelial cell membranes, (3) to endothelial cell membranes as well as accounting for capture by resting macrophages and (4) to endothelial cell membranes and accounting for capture by activated macrophages. To test the performance of our model, the corresponding Pearson's correlation coefficients [38] (denoted by *r*^2^) from the comparison of the model results with experiments across all organs are given in the centre of each panel. Additionally, in order to evaluate the performance of our model in predicting the targeted contributions, we have computed the correlation coefficient for the lung alone, as this organ consistently shows the effect due to targeting, and the corresponding *r*^2^ values are shown in the top left corner of the panels in figure 6*a*. Our results show that the targeting behaviour is well captured by our model for NC binding to endothelial cell membranes (*r*^2^∼0.88, panel (iii)) compared with the commonly used flat substrate models (*r*^2^∼0.57, panel (ii)). This quantitatively verifies the hypothesis that the mechanical properties of the endothelial cell membrane is an important contributor to NC targeting. The inclusion of additional contributions due to the macrophages does not alter the targeting behaviour in lung (panels (iv) and (v)) as the targeting component of the avidity is the dominant term for mouse lung tissues.

The inclusion of capture by other cells (e.g. macrophages or monocytes) actually worsens the prediction accuracy of NC targeting levels in all organs. The correlation coefficient computed across all organs is highest (*r*^2^=0.78) for the EC membrane model (i.e. without contribution from other cells). This implies that the non-specific uptake enhanced by EC capture is the dominant factor in differentiating the tissue targeting in the organs considered here. Hence, based on the statistical metrics we have presented in figure 6, we conclude that the model including the contributions from the endothelial cell membrane alone is the optimal choice in predicting NC targeting in mice tissues, and we use this model to make comparisons with other (additional) experiments (see below). We note that based on the above results, a very useful approximation for $\eta =({K_{p}K_{\mathrm{EC}}}_{\mathrm{org},\mathrm{sp}})/({K_{p}K_{\mathrm{EC}}}_{\mathrm{lung},\mathrm{sp}})$ holds; this implies that almost all of the conclusions we draw regarding the predictive power of our model are based only on two important parameters $K_{p}$ and *K*_EC_. The statistical performance of the various model scenarios including statistical significance of the model predictive power is summarized in table 3. The statistical procedures are explained in §2.6.

**Table 3.** Statistical testing of model scenarios: here ‘model’ refers to the NC targeting model and ‘null’ refers to model representing the null hypothesis (see §2); the comparison (*r*^2^) is between modeland experiment; ‘all’ refers to comparison across all organs and ‘lung’ refers to comparison for lung alone. (* indicates the activated form of cells.)

hypothesis	flat substrate	membrane	membrane + other cells	membrane + other cells*
*r*^2^: all/model	0.76 ± 0.003	0.77 ± 0.003	0.69 ± 0.005	0.64 ± 0.001
*r*^2^: all/null	0.59 ± 0.003	0.47 ± 0.001	0.37 ± 0.005	0.374 ± 0.003
*p*-value all	6×10^−13^	2×10^−14^	1×10^−15^	2×10^−12^
(model versus null)				
*r*^2^: lung/model	0.57 ± 0.009	0.88 ± 0.002	0.88 ± 0.002	0.88 ± 0.002
*r*^2^: lung/null	0.94 ± 0.003	0.61 ± 0.002	0.61 ± 0.002	0.61 ± 0.002
*p*-value lung	4×10^−07^	5×10^−7^	5×10^−7^	5×10^−7^
(model versus null)				

After having tested our model predictions against *in vivo* results for tissue targeting in mice, we now employ the model to make predictions for the tissue targeting of ICAM1-coated NCs in scenarios reported by several other studies [40,53–56]. The experimental values of *η*_exp_ are shown in figure 6*b* and, as noted earlier, the data show a large spread for spleen and liver. With the model parameters relevant for the tissue and NC in these experiments (see §2.7), we predict *η*_sim_, using equations (2.6) and (2.7), and the results are compared with *η*_exp_ in figure 6*c*. Our model predictions with zero-fitted parameters show good agreement with experimental results (*r*^2^=0.8), and points to the fact that our computational framework can give reliable and robust estimates for the tissue targeting of functionalized NC. The degree of agreement between our model predictions and six different sets of experimental results (figure 6) marks an important advance in the rational design of functionalized NC, with our proposed model framework providing a biophysical route for the optimization of functionalized NCs.

### 3.7 Predictions of tissue targeting of ICAM1-coated nanocarriers in human organs

**Figure 7.**
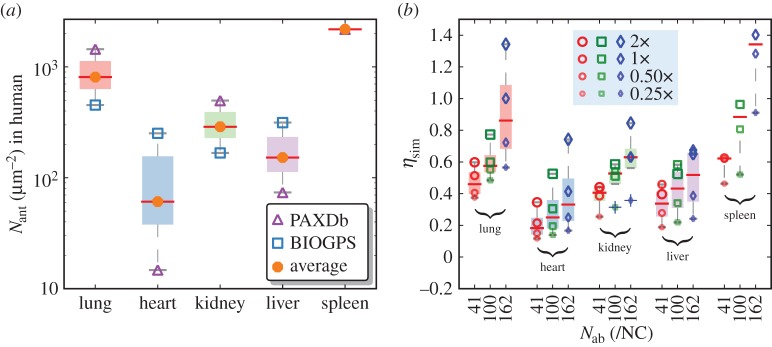
(*a*) Estimated expression levels of ICAM1 receptors, determined from PAXDb and BIOGPS. (*b*) Predictions for the efficacy of anti-ICAM1 NCs targeted to five different organs in human. Shown are the values of *η*_sim_ in lung, liver, heart, kidney and spleen for NCs with *N*_ab_=41, 100 and 162/NC. The various symbols show the sensitivity of *η*_sim_ when the receptor expression shown in panel (*a*) is modified by a factor of 0.25, 0.5, 1 or 2. In the whisker plot, the box represents the first quartile and the error bars span the entire spread of the data.

After having tested and shown the predictive capabilities of the computational framework using data from several tissue targeting experiments in mouse, we now proceed to make predictions for NC targeting in human organs. We treat the endothelial cells in the liver, lung, heart, kidney and spleen of humans to be mechanotypically (i.e. the values of the mechanical variables of the membrane) similar to the corresponding organs in mouse. The protein expression levels in the various organs are determined, as before, using data from high-throughput mRNA and mass spectrometry measurements in human tissue reported in the BIOGPS and PAXDb databases. We note that this estimate is based on the caveat that cellular regulation of the target protein expression and accessibility are often physiology- and pathology-dependent and are often not known, even though the corresponding data in mice showed excellent correlation between gene expression and target expression (figure 3). The density of ICAM1 receptors in the five organs of interest are shown in figure 7*a* and we find the expression levels to be the largest in the spleen (approx. 2500 ICAM1 μ*m*^−2^), while lung shows lower expression compared with that in mouse. The predictions for the targeting of anti-ICAM1-coated NCs, with *N*_ab_=41,100  162/NC, are shown in figure 7*b*. Our results for ICAM1-targeted carriers indicate that the NC tissue targeting in human lung shows characteristic targeting behaviour but its sensitivity is less pronounced compared with that seen in mouse (figure 6*a*). By contrast, NC tissue uptake in the spleen is much larger compared with lung, where a distinctive signature of targeting behaviour is also predicted by our model. In other organs, the evidence for any targeting is absent in our model predictions. Owing to the large discrepancy between PAXDb and BIOGPS data for heart and liver (figure 7*a*), we have also analysed the sensitivity of the tissue targeting levels by varying the expression levels of *N*_ant_ compared with that obtained using expression levels in mouse. We find that the lung and spleen shows a pronounced sensitivity to variations in the receptor expression levels compared with other organs.

## 4. Conclusion

Rational approaches in the design of functionalized NCs for targeted delivery can be immensely beneficial in optimizing the affinity of the NC in a tissue and species specific manner. We have presented a molecularly guided and biophysically based model framework to predict live-cell/tissue targeting of functionalized NCs across multiple organs and species. This framework is based upon a *zero-fit biophysically based multiscale model* to compute the binding avidity for an NC binding to a cell surface. In our model, we distinguish the various target endothelial cells in terms of their *mechanotype* (i.e. the mechanical properties of the cell membrane) and *phenotype* (i.e. the expression levels of the target protein). We also account for the contributions from *non-specific mechanisms in the tissue*. The model presented here combines the model-predicted estimates of the partitioning coefficients for affinity–ligand functionalized NCs in the target tissue and the experimentally determined partition coefficient for a non-targeted carrier of the same size and type, hence taking both the targeted and the non-targeted contributions to tissue targeting.

Our results emphasize the fact that the mechanotype and phenotype of the target cell are key parameters that can significantly influence NC binding, and hence should be integral to the design of functionalized NCs. We have used our computational framework to predict the tissue targeting levels of 100 nm anti-ICAM1 functionalized NCs in the lung, heart, liver, kidney and spleen of mouse and compared our findings to *in vivo* experiments, where available. Our results show that the targeting behaviour of anti-ICAM1 functionalized NCs in the mouse lung can be well captured only if the mechanical properties of the endothelial cell membrane and entropic effects coupled to multivalent binding are both explicitly taken into account. Predictions for other organs, which show characteristic non-targeted behaviour in *in vivo* experiments, also depend critically on target cell mechanotype and phenotype. Furthermore, we have also tested the performance of our model in predicting tissue targeting levels in the various scenarios reported in five different experiments available in the literature, and as demonstrated by the statistical metrics of the comparison, our predictions agree very well with the experimental findings. We have also demonstrated how our model predictions can be tailored to other organisms and organs by computing the targeting of ICAM1-coated NCs in conditions that mimic human tissues. It should be noted that in terms of parameter determination, there is a larger variation between the physically based antibody-tracing method and other indirect methods of ICAM1-expression determination in spleen and liver than in the lungs. This suggests that the relative contribution of ICAM1 in the vascular lumen relative to the other compartments is organ specific. Hence, experiments directly mapping ICAM1 accessibility rather than indirectly determining gene or protein expression may be even more important for better characterization of the tissue microenvironment.

One of the limitations of our current model is that it only explores steady-state behaviour at timescales when internalization mediated by ICAM1 has not set in. We also note that our predictions rely on the accurate quantification of the ‘non-targeted’ capture (i.e. not directly mediated by ICAM1 binding) contributions through experiments since predictively accounting for such contributions is beyond the scope of the current model. Non-specific factors such as clearance mechanisms can be different for targeted and non-targeted uptake. The different cell types (including and beyond the endothelial cells and macrophages) in the tissue microenvironment can sustain context-dependent uptake mechanisms such as ICAM1- and FC receptor-mediated endocytosis, or macropinocytosis. Other physiological and haemodynamic factors such as vascular hydrodynamics and NC margination [16,64], glycocalyx [13,20], cytoskeletal dynamics and NC internalization [36,37] are also physiology- and pathology-dependent. These considerations warrant more work and will be addressed in the future through which we expect a significant improvement in *r*^2^ between model and experiment. Future extensions of the next-generation PK model presented here will also focus on predictively modelling the targeting of other epitopes in the vasculature (such as PECAMs), modelling targeting of epitopes in epithelial tissues and predictive modelling of non-targeted contributions discussed above.

## Supplementary Material

Supplementary Information

## Data accessibility

Sections S1–S4 contain detailed description of the computational model and derivation of the association constant and standardized uptake values. Movies M1–M7 show the binding of an NC to the seven different cell membranes shown in figure 4. These are available online under Supplementary Information. Access to raw data and codes can be requested through email to the corresponding author.
