# Supplementary material for: Biophysically inspired model for functionalized nanocarrier adhesion to cell surface: roles of protein expression and mechanical factors
Source: R Soc Open Sci. 2016 Jun 29;3(6):160260. doi: 10.1098/rsos.160260 (PMC4929918; doi:10.1098/rsos.160260)
Supplement: Supplementary Information [file rsos160260supp1.pdf]

Supplementary Information — Biophysically inspired model for functionalized nanocarrier adhesion to cell surface: roles of protein expression and mechanical factors

N. Ramakrishnan[1], R. W. Tourdot[6], David M. Eckmann[1,2], Portnovo S. Ayyaswamy[3], Vladimir R. Muzykantov[4,5], and Ravi Radhakrishnan[1,6,7]

[1] *Department of Bioengineering, University of Pennsylvania, Philadelphia, PA, 19104, USA*

[2] *Department of Anesthesiology and Critical Care, University of Pennsylvania, Philadelphia, PA, 19104, USA*

[3] *Department of Mechanical engineering and Applied Mechanics, University of Pennsylvania, Philadelphia, PA, 19104, USA*

[4] *Center for Targeted Therapeutics and Translational Nanomedicine, Institute for Translational Medicine & Therapeutics and Department of Pharmacology, University of Pennsylvania School of Medicine, Philadelphia, PA, 19104, USA*

[5] *Translational Research Center, University of Pennsylvania, The Perelman School of Medicine, Philadelphia, PA, 19104, USA*

[6] *Department of Chemical and Biomolecular engineering, University of Pennsylvania, Philadelphia, PA, 19104, USA and*

[7] *Department of Biochemistry and Biophysics, University of Pennsylvania, Philadelphia, PA, 19104, USA*

S1. MONTE CARLO TECHNIQUES FOR NANOCARRIER-MEMBRANE SIMULATIONS

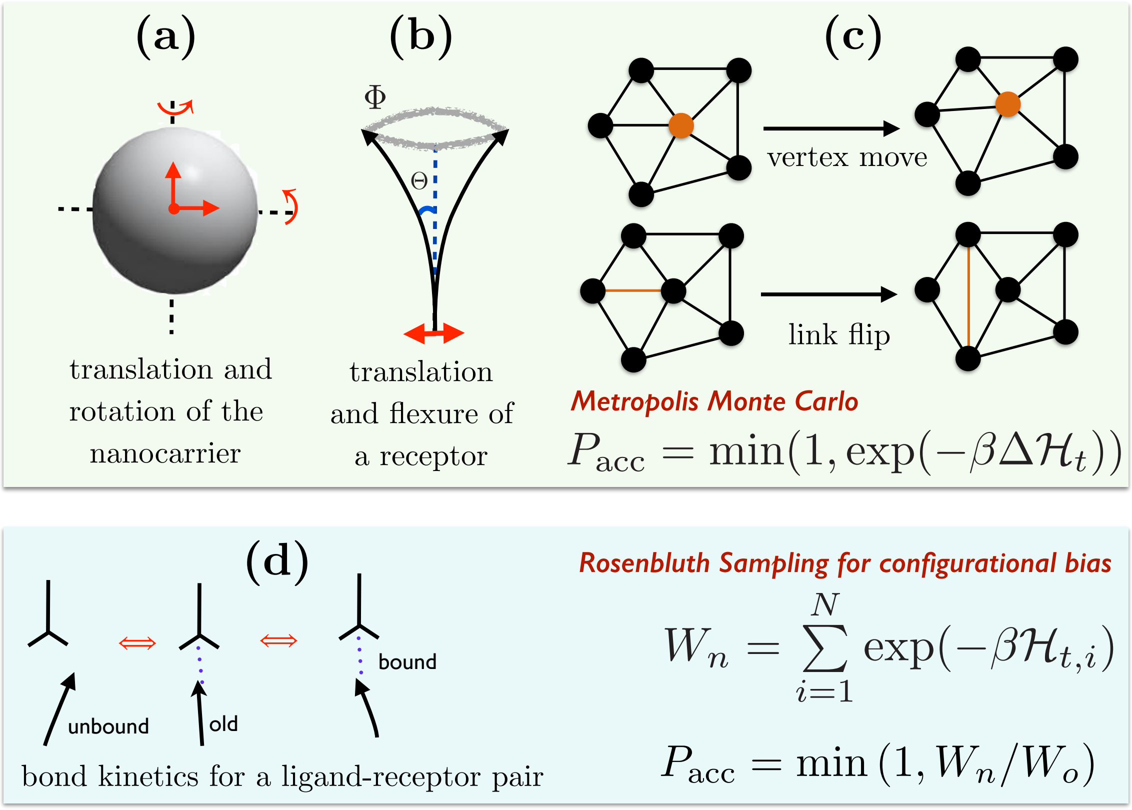

Figure S1. An illustration of the various Monte Carlo moves to evolve the thermodynamic state of the membrane-nanocarrier system. (a) Random translational and rotational moves of the nanocarrier, (b) diffusion and flexure of the receptors, (c) dynamically triangulated Monte Carlo moves for the membrane, and (d) Rosenbluth sampling based configurational bias for formation and breakage of receptor-ligand bonds.

Fig. S1 shows the seven different Monte Carlo moves to evolve microstates of the nanocarrier (NC)-membrane system:

(1) *NC translation*: An attempt to randomly displace the center of mass of the NC within a cube of length δ centered around its current position. The value of δ is chosen (and also modifed during runtime) such that nearly 50% are the attempted moves are accepted [1, 2].

(2) *NC rotation*: An attempt is made to change the orientation of the nanocarrier from

$\{\theta, \phi, \psi\} \rightarrow \{\theta + \Delta\theta, \phi + \Delta\phi, \psi + \Delta\psi\}$ where $\{\theta, \phi, \psi\}$ are the three Euler angles $\{\Delta\theta, \Delta\phi, \Delta\psi\}$ are random numbers chosen from an uniform distribution in the range $[0, 2\pi]$ (for $\Delta\theta$ and $\Delta\psi$) or $[0, \pi]$ (for $\Delta\phi$) [1, 2].

(3) *Translation of receptors*: An attempt is made to randomly displace a receptor molecule to a new position on the membrane surface [1, 2].

(4) *Flexure of receptors* : An attempt is made to change the flexure angles of the receptor from $\{\Theta, \Phi\} \rightarrow \{\Theta + \Delta\Theta, \Phi + \Delta\Phi\}$, where $\Delta\Theta$ is drawn from a distribution $\sin^{-1}([-1.0, 1.0])$ and $\Delta\Phi$ is drawn from an uniform distribution in the interval $[0, 2\pi]$ [1, 2].

(5) *Vertex move*: A random chosen vertex on the triangulated surface is displaced to a new position (as shown in Fig. S1(c)) holding the triangulation fixed [3, 4]

(6) *Link flip* : A link shared by two neighboring faces of the triangulated surface is removed and reconnected to the two previously unconnected vertices as illustrated in Fig. S1(c) [3, 4].

Moves (1)-(6) fall under the class of canonical Monte Carlo and hence are accepted using a Metropolis scheme [5] such that $P_{\text{acc}} = \min\left(1, \exp(-\beta\Delta\mathcal{H}_t)\right)$

(7) *Formation and breakage of receptor-ligand bonds* : The formation of a receptor-ligand bonds is very sensitive to the conformations of the membrane and position of the nanocarrier and hence can be treated as a rare event compared to moves (1)-(6). In order to exhaustively sample the bonded states of the system, we use the Rosenbluth sampling technique [6] to sample bond formation and breakage moves. The attempted move is accepted according to $P_{\text{acc}} = \min(1, W_n/W_o)$. Here $W_n = \sum_{i=1}^{N} \exp(-\beta\mathcal{H}_{t,i})$ is the weight factor to generate N independent configurations with energy $\mathcal{H}_{t,i}$ and $W_o = \exp(-\beta\mathcal{H}_{t,*}) + \sum_{i=2}^{N} \exp(-\beta\mathcal{H}_{t,i})$ is the weight factor to generate $N-1$ independent configurations starting with the initial configuration whose energy is given by $\mathcal{H}_{t,*}$.

S2. ASSOCIATION CONSTANT FOR A NANOCARRIER BOUND TO A CELL MEMBRANE

Consider a spherical nanocarrier of radius a, expressing $N_{\rm ab}$ antibodies on its surface, and is bound to a cell membrane, expressing $N_{\rm ant}$ surface receptors. If $\mathbf{X}$, $\mathbf{M}$, and $\mathbf{R}$ be the shorthand notations for the nanocarrier, membrane, and receptor degrees of freedom, then we define the configurational integrals for the individual components as

$$\int d\mathbf{X} = \int dxdydz \int_0^{2\pi} d\phi \int_0^{\pi} \sin\theta \, d\theta \int_0^{2\pi} d\psi, \tag{1}$$

where x, y, and z denote the Cartesian coordinate system and ϕ, θ, and ψ denote the three Euler angles. The above equation describes the volume accessible to an unbound nanocarrier in the configurational space and can further be reduced to,

$$\int d\mathbf{X} = 8\pi^2 \int dxdydz. \tag{2}$$

If R_i denotes the position vector of the i th receptor molecule, the configurational integral for the receptor degrees of freedom may be expressed as,

$$\int d\mathbf{R} = \prod_{i=1}^{N_{\rm ant}} \int dR_i, \tag{3}$$

and if M_i denote the three dimensional position of the i th coarse grained bead on the membrane (i.e., the i th vertex of the triangulated surface) the configurational space for the membrane is defined as

$$\int d\mathbf{M} = \prod_{i=1}^{N_{\rm ant}} \int dM_i. \tag{4}$$

Following [7, 8] the probability for the nanocarrier to be in an unbound state (i.e., multivalency $m = 0$) as,

$$\mathcal{P}_0 = \int d\mathbf{X} \int d\mathbf{R} \int d\mathbf{M} \exp(-\beta \mathcal{H}_{\rm sur}). \tag{5}$$

If we treat the membrane surface to be a flat substrate, its energy $\mathcal{H}_{\rm sur} = 0$ and the contribution from the membrane degrees of freedom can be taken to be a constant, which allows us rewrite the above equation as,

$$\mathcal{P}_0 = \int_{r^*}^{L_z} d\mathbf{X} \int_u d\mathbf{R} = 8\pi^2 \mathcal{A}_N^u (L_z - r^*)(\mathcal{A}_R^u)^{N_{\rm ant}}. \tag{6}$$

Here the integrations are carried out over all the unbounded states, $\mathcal{A}_N^u$ and $\mathcal{A}_R^u$ are the areas traversed by the nanocarrier and the receptor molecules, respectively, in their unbound state, and r^* denotes the upper limit for the reaction coordinates beyond which bond formation is disallowed purely for geometric reasons. Now, consider the formation of the first receptor-ligand bond which is penalized by an energy cost given by the potential of mean force $\mathcal{W}(r)$, where r denotes a reaction coordinate. Since any given receptor molecule can in principle form bonds with any given antibody on the nanocarrier surface, the total number of microstates with a single receptor-ligand bond is given by $N_{\rm ab} \times N_{\rm ant}$ and the probability to find the nanocarrier with a multivalency of 1 can be expressed as,

$$\mathcal{P}_1 = \underbrace{N_{\rm ab} N_{\rm ant}}_{permutations} \underbrace{\int_{\phi_0}^{\phi_0+\delta\phi} d\phi \int_{\theta_0}^{\theta_0+\delta\theta} \sin\theta d\theta \int_{\psi_0}^{\psi_0+\delta\psi} d\psi}_{rotational\ \ volume} \underbrace{\int_b dR_1}_{\mathcal{A}_R^b} \underbrace{\int_u dR_2 \cdots dR_{N_{\rm ant}}}_{\mathcal{A}_R^u \times \cdots \times \mathcal{A}_R^u} \mathcal{A}_N^b \int_0^{r^*} dr \exp(-\beta \mathcal{W}(r)), \tag{7}$$

where the orientation of the nanocarrier is constrained to be fluctuating around the Euler angles ϕ_0, θ_0, and ψ_0, when a specific antibody is engaged in bonding. $\mathcal{A}_R^b$ and $\mathcal{A}_N^b$ are the area traversed by a bound receptor and the center of mass of the bound nanocarrier. The above equation can be generalized to n_b receptor-ligand bonds as,

$$\mathcal{P}_{n_b} = \binom{N_{\rm ant}}{n_b} \frac{N_{\rm ab}}{n_b} (\Delta\phi\, \Delta\theta\, \Delta\psi)\, (\mathcal{A}_R^b)^{n_b} (\mathcal{A}_R^u)^{N_{\rm ant}-n_b} \mathcal{A}_N^b \int_0^{r^*} dr \exp(-\beta \mathcal{W}(r)), \tag{8}$$

As pointed in out in [7, 8], the reversible work done to go from a unbounded state (with $n_b = 0$) to state with n_b simultaneous receptor-ligand bonds is the logarithm of the ratio,

$$\frac{\mathcal{P}_{n_b}}{\mathcal{P}_0} = \binom{N_{\rm ant}}{n_b} \frac{N_{\rm ab}}{n_b} \frac{(\Delta\phi\, \Delta\theta\, \Delta\psi)}{8\pi^2} \left(\frac{\mathcal{A}_R^b}{\mathcal{A}_R^u}\right)^{n_b} \frac{\mathcal{A}_N^b}{\mathcal{A}_N^u (L_z - r^*)} \int_0^{r^*} dr \exp(-\beta \mathcal{W}(r)), \tag{9}$$

which is also related to the association constant as,

$$K_a = \frac{1}{[L]} \frac{\mathcal{P}_{n_b}}{\mathcal{P}_0}, \tag{10}$$

with the nanocarrier concentration $[L] = (\mathcal{A}_N^u L_z)^{-1}$ for a system with exactly one nanocarrier. Using eqn. (9) in eqn. (10) we get the final expression for the association constant

to be,

$$K_a = \underbrace{\left(\frac{N_{\text{ant}}!}{(N_{\text{ant}} - n_b)!\, n_b!}\right)\binom{N_{\text{ab}}}{n_b}}_{\substack{\text{combinatorial entropy}\\ \text{for receptor-ligand bond formation}}} \underbrace{\left(\frac{\Delta\phi\, \Delta\theta\, \Delta\psi}{8\pi^2}\right)}_{\substack{\text{NC rotational}\\ \text{entropy}}} \underbrace{\left(\frac{\mathcal{A}_R^b}{\mathcal{A}_R^u}\right)^{n_b}}_{\substack{\text{translational entropy}\\ \text{of receptors}}} \underbrace{\frac{\mathcal{A}_N^b L_z}{(L_z - r^*)} \int_0^{r^*} dr \exp(-\beta \mathcal{W}(r))}_{\text{enthalpic contribution}}. \tag{11}$$

a. *Computing spatial maps for the bound receptors:* The spatial map of the bound receptors, for a given conformation of the membrane and the nanocarrier, is computed as follows. The mean orientation of the bound receptor-ligand bonds $\hat{\mathbf{n}}$ defines a tangent plane that is shown in Fig. S2. A vector in the cartesian coordinate system can be projected onto this tangent plane using the projection operator $\mathbb{P} = \mathbb{I} - \hat{\mathbf{n}} \otimes \hat{\mathbf{n}}$, where $\mathbb{I}$ is the unit matrix. We project the coordinates of the nanocarrier and the position of

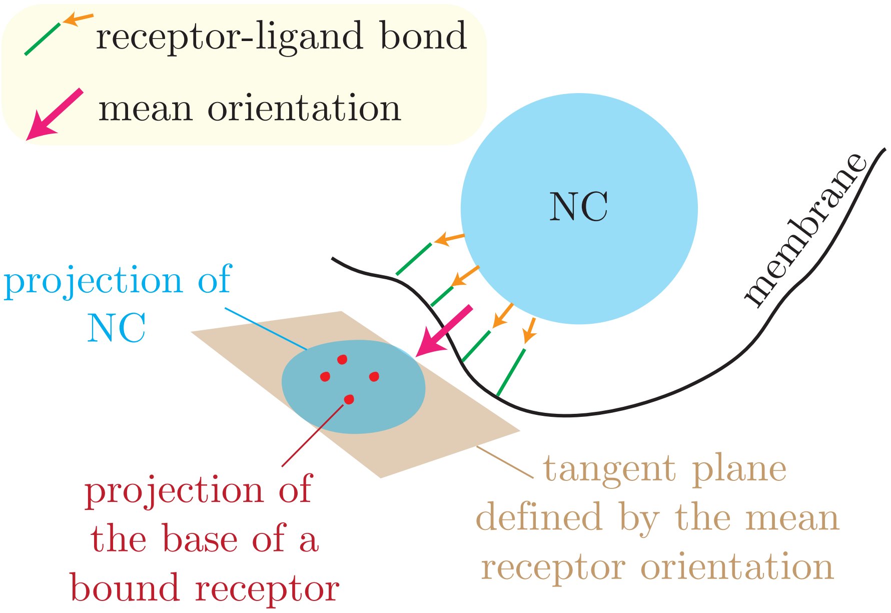

Figure S2. An illustration of a nanocarrier bound to a membrane through multiple receptor-ligand bonds. The mean orientation of the bound receptor-ligand bonds defines a tangent plane and the projections of the NC and the bound receptors onto the tangent plane are also shown.

the bound ligands onto this plane, as shown in Fig. S2, to obtain spatial maps which are used to estimate the area traversed by a bound nanocarrier, and a bound receptor. We represent these patterns in terms of $[\hat{t}_1, \hat{t}_2]$, the orthonormal unit vectors in a coordinate system attached to this plane.

S3. FORMULATION OF THE PHARMACO-KINETIC (PK) MODEL: RELATION TO STANDARDIZED UPTAKE VALUES

Conventional PK model for non-targeted delivery: In classical perfusion limited pharmacokinetic models [9, 10], with C_T denotes the concentration of the drug in a tissue compartment whose volume is V_T, the outlet concentration C_{out} at steady-state is expressed as:

$$C_T = \mathcal{K}_p C_{out}, \tag{12}$$

with $\mathcal{K}_p$ being the partitioning coefficient which denotes the ratio of the probability for the drug to be associated with the tissue to that in the blood stream.

A. Targeted uptake

In considering NC targeting, the tissue volume is divided into two compartments, namely the endothelial cells lining the vasculature and the bulk tissue. The NC concentration at the endothelial cell surface and in the tissue are given by C^* and C_T, as shown in Fig. S3. As before, the volume of the tissue is V_T.

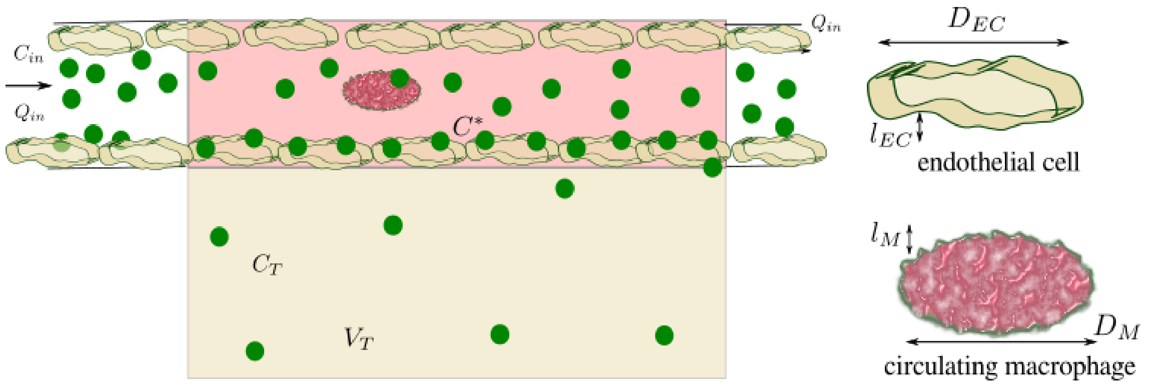

Figure S3. A schematic for the control volume of the tissue and the endothelial cells lining its interface with the blood vessels.

In the absence of any functional groups the injected NCs are absorbed from the blood stream and distributed into the tissue primarily through non-specific mechanisms such as diffusion, permeation, and facilitated transport, that proceed via both intracellular and extracellular routes. In such a scenario, the concentration of the nanoparticles on the endothelial surface is given by $C^* = C_{out}$.

Targeted NCs, on the other hand, adhere preferentially to the endothelial cells and as a result the concentration of the nanoparticle at the boundary of the tissue differs from C_{out}. The concentration of the NC is enhanced at the endothelial cells boundaries such that the enhanced value $C^* \neq C_{out}$, and in this case the partitioning of the NC concentration in the tissue is given by:

$$C_T = \mathcal{K}_p C^*. \tag{13}$$

In order to proceed with the development of the PK model for targeted carriers, it is necessary to establish a relation between C^* and the bulk concentration C_{out}. It should be noted that C^* is the concentration of the NCs in the endothelial cells computed with respect to the volume of the tissue V_T. Hence, the total number of NCs bound to an endothelial cell (with cross sectional area l_{EC}^2), when the NC concentration is C^*, is:

$$N^{bound} = C^* l_{EC}^2 \mathcal{L}_{EC,b}, \tag{14}$$

where $\mathcal{L}_{EC,b}$ is the length of the boundary layer within which the NC concentration is enhanced due to binding; i.e., $\mathcal{L}_{EC,b} = r^*$ in Eq. 11 for EC. N^{bound} can also be expressed as $C_{out} l_{EC}^2 L_{cap} P_b$, where L_{cap} is the size of the cell free layer in the capillary in which the NC marginates and P_b is the probability of NC binding. Using the relationship for P_b given by [11]:

$$K_{EC} C_{out} = \frac{P_b}{1 - P_b}, \tag{15}$$

or alternatively,

$$P_b = \frac{K_{EC} C_{out}}{(1 + K_{EC} C_{out})}, \tag{16}$$

we obtain in the limit of $K_{EC} C_{out} \ll 1$,

$$C^* = \frac{L_{cap}}{\mathcal{L}_{EC,b}} K_{EC} C_{out}^2. \tag{17}$$

Assuming that the entire NC fraction bound to the EC ends up in the tissue, (i.e., we assume the NC enternalization is neither rate-limiting nor saturates at steady state, and that the bound NC fraction on the EC also contributes to the tissue targeting *in vivo*), the concentration of the NC which includes contributions from both the tissue and from those adhered to the endothelial cells needs to be accounted for in the biodistribution. Hence, the total number of nanoparticles harvested in the tissue N_{tot} is given by:

$$N_{tot} = N_T + N_{EC} = \mathcal{K}_p C^* + C^* l_{EC}^2 \mathcal{L}_{EC,b}, \tag{18}$$

Experiments measure the volume concentration of endothelial cells as $\varphi_{EC} = V_{EC}/V_T$, where the endothelial cell volume is approximated as $V_{EC} = l_{EC}^2 D_{EC}$ with D_{EC} being the EC diameter. The above equation may be rewritten as:

$$C_{tot} = \frac{N_{tot}}{V_T} = \{\mathcal{K}_p K_{EC} C_{out}^2 + \frac{\varphi_{EC}\, K_{EC}\, \mathcal{L}_{EC,b}}{D_{EC}}\, C_{out}^2\} \times \frac{L_{cap}}{\mathcal{L}_{EC,b}}. \tag{19}$$

This framework can also account for additional contributions such as the targeted uptake of nanoparticles by other circulating cells (such as monocytes etc.). As before, if $\mathcal{L}_{M,b}$ denotes the length of the boundary layer in which NC concentration is enriched on the surface of the other cells, D_M the diameter, and φ_M the volume concentration of other cells, then the expression for the total concentration of NCs is given by,

$$C_{tot} = \{\mathcal{K}_p K_{EC} C_{out}^2 + \frac{\varphi_{EC}\, K_{EC}\, \mathcal{L}_{EC,b}}{D_{EC}}\, C_{out}^2\} \times \frac{L_{cap}}{\mathcal{L}_{EC,b}} + \frac{\varphi_M\, K_M\, \mathcal{L}_{M,b}}{D_M}\, C_{out}^2 \times \frac{L_{cap}}{\mathcal{L}_{M,b}}, \tag{20}$$

where, K_M is the association constant for targeted binding of nanoparticles to other cells.

The above equation can be related to the standardized uptake value, usually measured in percentage injected dose per gram of tissue, as

$$\%idg \sim \frac{C_{tot}}{C_{out}} = \{\mathcal{K}_p K_{EC} C_{out} + \frac{\varphi_{EC}\, K_{EC}\, \mathcal{L}_{EC,b}}{D_{EC}}\, C_{out}\} \times \frac{L_{cap}}{\mathcal{L}_{EC,b}} + \frac{\varphi_M\, K_M\, \mathcal{L}_{M,b}}{D_M}\, C_{out} \times \frac{L_{cap}}{\mathcal{L}_{M,b}}, \tag{21}$$

Experiments based on targeting specific receptors have shown that other cells (e.g., monocytes) and endothelial cells are found with nearly similar compositions in a tissue, with typical values to be around $\varphi_{EC} \sim 3-30\%$, $\varphi_M \sim 3-10\%$ [12–14]. The references [15, 16] provide estimates of the diameters of endothelial cells and other cells.

S4. SUPPLEMENTARY MOVIES

.

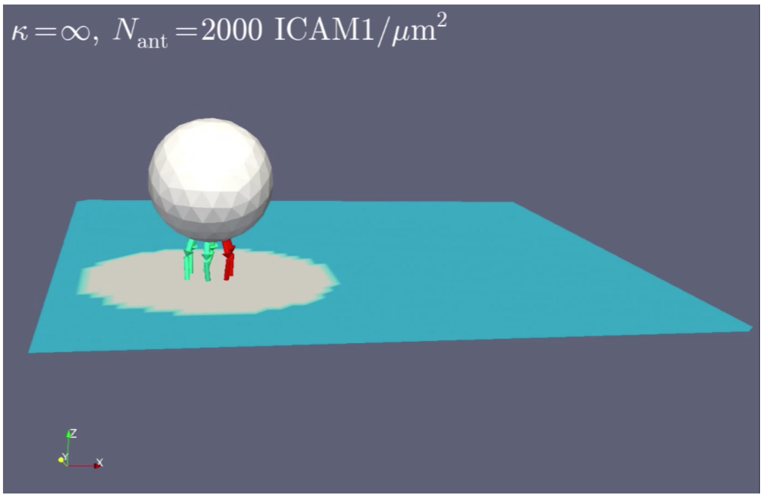

Figure S4. M1: Movie of an NC, with $N_{\mathrm{ab}} = 162$, bound to a cell membrane with $\kappa = \infty$, $\mathcal{A}_{\mathrm{ex}} = 0\%$, and N_{ant}= 2000 ICAM1/μm^2.

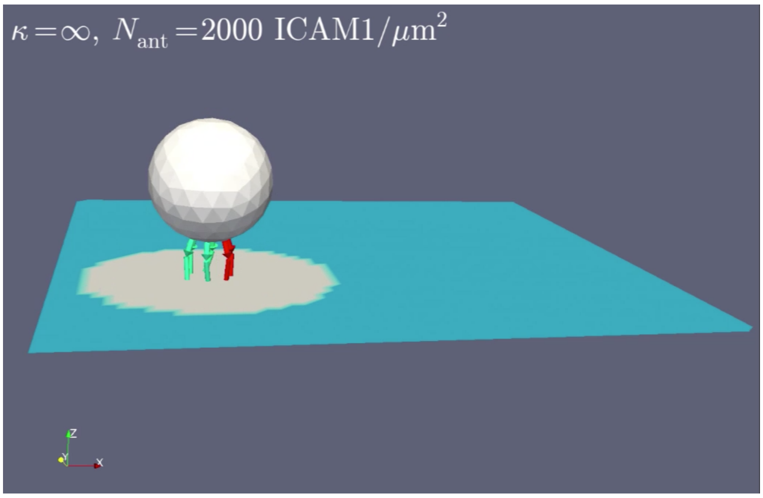

Figure S5. M2: Movie of an NC, with $N_{\mathrm{ab}} = 162$, bound to a cell membrane with $\kappa = 160\, k_{\mathrm{B}}T$, $\mathcal{A}_{\mathrm{ex}} \sim 2\%$, and N_{ant}= 2000 ICAM1/μm^2.

---

[1] N. J. Agrawal and R. Radhakrishnan, J. Phys. Chem. C **111**, 15848 (2007).

[2] J. Liu, G. E. Weller, B. Zern, P. S. Ayyaswamy, D. M. Eckmann, V. R. Muzykantov, and R. Radhakrishnan, Proc. Natl. Acad. Sci. U.S.A. **107**, 16530 (2010).

[3] J. S. Ho and A. Baumgärtner, Euro. Phys. Lett. **12**, 295 (1990).

[4] N. Ramakrishnan, P. B. Sunil Kumar, and J. H. Ipsen, Phys. Rev. E **81**, 041922 (2010).

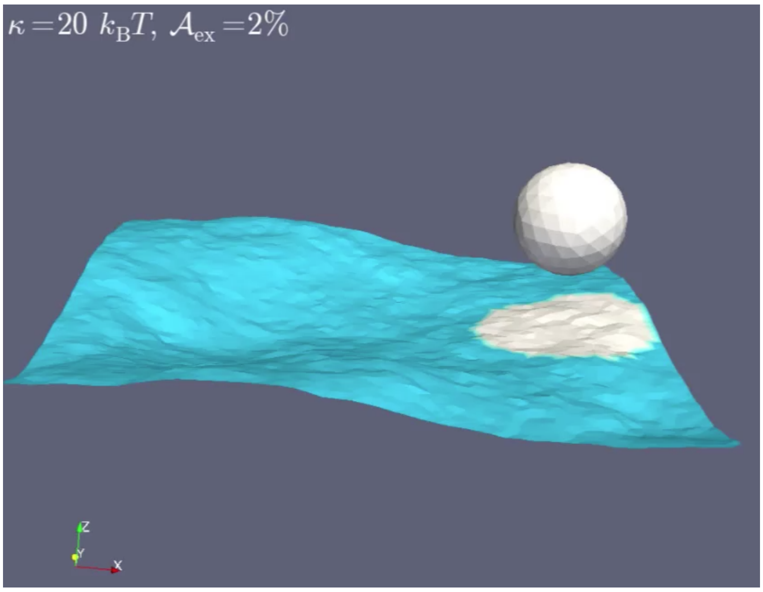

Figure S6. M3: Movie of an NC, with N_{ab} = 162, bound to a cell membrane with $\kappa = 20\,k_BT$, $\mathcal{A}_{ex} \sim 3\%$, and N_{ant}= 2000 ICAM1/μm^2.

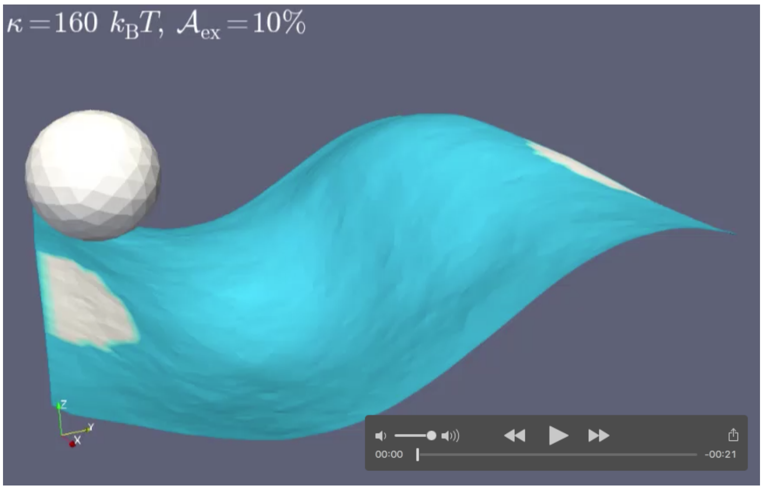

Figure S7. M4: Movie of an NC, with N_{ab} = 162, bound to a cell membrane with $\kappa = 160\,k_BT$, $\mathcal{A}_{ex} \sim 14\%$, and N_{ant}= 2000 ICAM1/μm^2.

[5] N. Metropolis, A. W. Rosenbluth, M. N. Rosenbluth, A. H. Teller, and E. Teller, J. Chem. Phys. **21**, 1087 (1953).

[6] D. Frenkel and B. Smit, *Understanding Molecular Simulation : From Algorithms to Applications*, 2nd ed. (Academic Press, 2001).

[7] B. Roux, M. Nina, R. Pomes, and J. C. Smith, Biophys. J. **71**, 670 (1996).

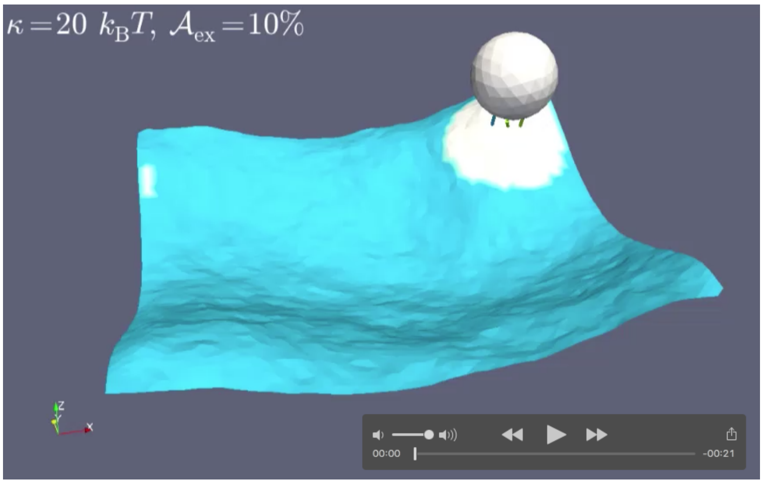

Figure S8. M5: Movie of an NC, with $N_{ab} = 162$, bound to a cell membrane with $\kappa = 20\, k_B T$, $\mathcal{A}_{ex} \sim 10\%$, and N_{ant}= 2000 ICAM1/μm^2.

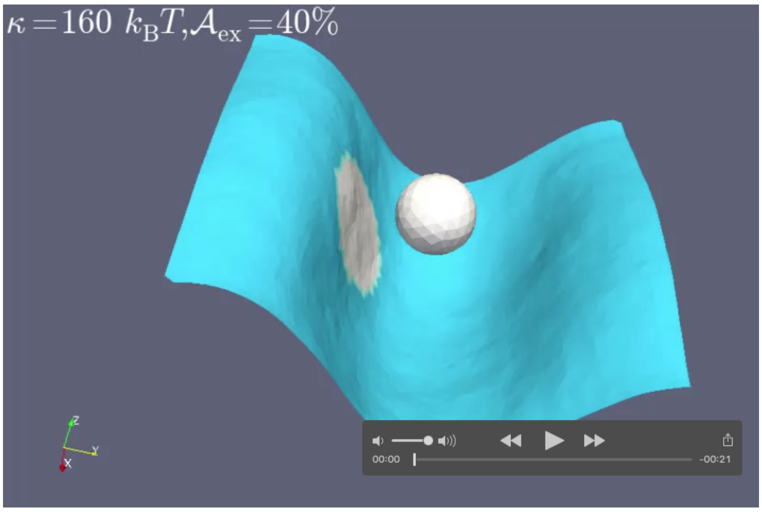

Figure S9. M6: Movie of an NC, with $N_{ab} = 162$, bound to a cell membrane with $\kappa = 160\, k_B T$, $\mathcal{A}_{ex} \sim 37\%$, and N_{ant}= 2000 ICAM1/μm^2.

[8] H.-J. Woo and B. Roux, Proc. Natl. Acad. Sci. U.S.A. **102**, 6825 (2005).

[9] I. Nestorov, Clin Pharmacokinet **42**, 883 (2003).

[10] M. Li, K. T. Al-Jamal, K. Kostarelos, and J. Reineke, ACS Nano **4**, 6303 (2010).

[11] K. A. Dill and S. Bromberg, *Molecular Driving Forces*, 2nd ed. (Garland Science, New York, 2011).

[12] J. D. Crapo, B. E. Barry, P. Gehr, M. Bachofen, and E. R. Weibel, Am. Rev. Respir. Dis. **125**, 740 (1982).

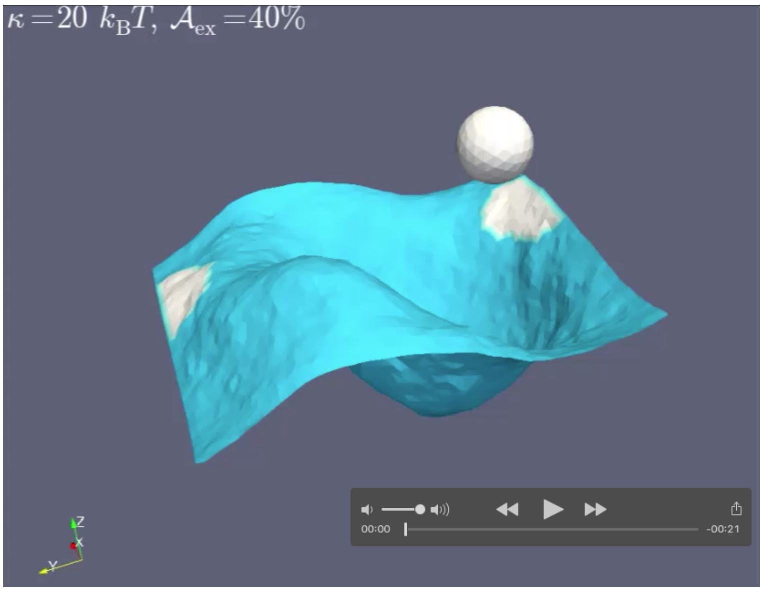

Figure S10. M7: Movie of an NC, with $N_{ab} = 162$, bound to a cell membrane with $\kappa = 20\, k_BT$, $\mathcal{A}_{ex} \sim 44\%$, and N_{ant}= 2000 ICAM1/μm^2.

[13] S. H. Lee, P. M. Starkey, and S. Gordon, J. Exp. Med. **161**, 475 (1985).

[14] J. R. van Beijnum, M. Rousch, K. Castermans, E. van der Linden, and A. W. Griffioen, Nature Protocols **3**, 1085 (2008).

[15] K. A. Barbee, P. F. Davies, and R. Lal, Circulation Research **74**, 163 (1994).

[16] B. Garipcan, S. Maenz, T. Pham, U. Settmacher, K. D. Jandt, J. Zanow, and J. Bossert, Adv. Eng. Mater. **13**, B54 (2010).
